# Transcriptional Dynamics Elicited by a Short Pulse of Notch Activation Involves Feed-Forward Regulation by *E(spl)/Hes* Genes

**DOI:** 10.1371/journal.pgen.1003162

**Published:** 2013-01-03

**Authors:** Ben E. Housden, Audrey Q. Fu, Alena Krejci, Fred Bernard, Bettina Fischer, Simon Tavaré, Steven Russell, Sarah J. Bray

**Affiliations:** 1 Department of Physiology, Development, and Neuroscience, University of Cambridge, Cambridge, United Kingdom; 2 Cambridge Systems Biology Centre, University of Cambridge, Cambridge, United Kingdom; 3 Department of Genetics, University of Cambridge, Cambridge, United Kingdom; 4 Department of Applied Mathematics and Theoretical Physics, University of Cambridge, Cambridge, United Kingdom; Cancer Research UK London Research Institute, United Kingdom

## Abstract

Dynamic activity of signaling pathways, such as Notch, is vital to achieve correct development and homeostasis. However, most studies assess output many hours or days after initiation of signaling, once the outcome has been consolidated. Here we analyze genome-wide changes in transcript levels, binding of the Notch pathway transcription factor, CSL [Suppressor of Hairless, Su(H), in *Drosophila*], and RNA Polymerase II (Pol II) immediately following a short pulse of Notch stimulation. A total of 154 genes showed significant differential expression (DE) over time, and their expression profiles stratified into 14 clusters based on the timing, magnitude, and direction of DE. *E(spl)* genes were the most rapidly upregulated, with Su(H), Pol II, and transcript levels increasing within 5–10 minutes. Other genes had a more delayed response, the timing of which was largely unaffected by more prolonged Notch activation. Neither Su(H) binding nor poised Pol II could fully explain the differences between profiles. Instead, our data indicate that regulatory interactions, driven by the early-responding *E(spl)bHLH* genes, are required. Proposed cross-regulatory relationships were validated *in vivo* and in cell culture, supporting the view that feed-forward repression by *E(spl)bHLH/Hes* shapes the response of late-responding genes. Based on these data, we propose a model in which *Hes* genes are responsible for co-ordinating the Notch response of a wide spectrum of other targets, explaining the critical functions these key regulators play in many developmental and disease contexts.

## Author Summary

Signaling via the Notch pathway conveys important information that helps to shape tissues and, when misused, contributes to diseases. Cells respond to the Notch signal by changing which genes are transcribed. Most previous studies have looked at changes in gene activity at a single time point, long after the start of signaling. By looking at carefully timed intervals immediately after Notch pathway activation, we have been able to follow the dynamic changes in transcription of all the genes and have found that they exhibit different patterns of activity. For example, activity of some genes, especially a previously characterised family called the *E(spl)* genes, starts very early, whereas others show more delayed upregulation. Our investigations into the underlying mechanisms reveal that cross-regulatory interactions driven by the early genes are required to shape the timing of the delayed response. This feed-forward mechanism is important because it explains why the *E(spl)/Hes* genes can play such a pivotal role in the Notch response, despite the fact that many other genes are regulated by the signal, a finding that will be valuable for understanding the contribution of *E(spl)/Hes* genes in diseases associated with altered Notch.

## Introduction

Cell fates during development are controlled, in part, by the activity of signaling pathways including Notch. Frequently, the response to signaling pathways is analyzed many hours or days after signaling is initiated, despite the fact that early events can occur within seconds of the stimulus and transcriptional changes can arise within minutes [1]–[4]. Gaining insights into the immediate changes that take place in response to signaling activity is therefore of major importance in understanding the regulatory mechanisms that ultimately translate into fate decisions. For example, crosstalk between growth factor signaling and centrosome assembly was revealed by analyzing ubiquitinylation within 30 minutes of stimulation [1].

A crucial step in signaling pathway function is transmission of the signal to the nucleus. For many pathways there are multiple intermediate steps in this relay. However the activation of Notch has a direct route to the nucleus as it elicits a proteolytic cleavage that releases the Notch intracellular domain (N^icd^). This directly enters the nucleus to generate a rapid transcriptional response [5]–[9]. Real time imaging indicates translocation occurring within minutes of activation [2], [3]. Once in the nucleus, N^icd^ associates with transcription factors of the CSL family (Su(H) in *Drosophila*) [8], [9]. Thus, effects on gene expression are a primary consequence of activating the pathway, making it important to analyse the transcriptional changes that are elicited as well as the ultimate changes in cell fate.

The Notch pathway functions in many different developmental decisions, in some cases preventing differentiation and in others promoting it [10]–[12]. Inappropriate activity of Notch also contributes to many diseases including cancers, where it promotes or prevents tumorigenesis depending on the context [11], [13]–[16]. Despite these differences, genes of the Hairy and Enhancer of split family (*Hes* genes) are strongly upregulated in the majority of Notch signaling contexts that have been analysed [17]–[20]. These genes encode bHLH repressors that are direct targets of the Notch pathway and their expression can account for much of the functional output of the pathway. For example, during neurogenesis their inhibition of proneural proteins is critical [21], [22] and in T-cell acute lymphoblastic leukaemia their repression of PTEN contributes to the cancer [23], [24]. Thus, mutations eliminating *Hes* gene function frequently phenocopy the loss of Notch [18], [25]–[28]. However, more recently it has become evident that there are complex programmes of gene expression changes elicited by Notch activation. Such results have emerged principally from analysis of genome-wide changes in transcription that are detected in cell culture using simple strategies to manipulate Notch activity [29]–[32]. The hundreds or thousands of genes whose expression has been found to change following Notch signaling are hard to reconcile with the previous evidence demonstrating the importance of the *Hes* genes. Furthermore, the targets frequently include positive and negative regulators of the same pathways, making the logic of the response hard to interpret [29], [33].

The discovery of these large and diverse cohorts of Notch targets raises the possibility that different targets are activated with different kinetics and/or at different levels of signaling. If this were the case it could have profound effects on the outcome [34]. It would also raise the question of how, mechanistically, such differential responses could be generated. One possibility is that target genes contain different numbers or arrangements of CSL binding sites [35]–[40]. Another is that some genes may be primed for activation by the prior recruitment of Polymerase II (Pol II) [41]–[44]. Elucidating the temporal and kinetic relationships between target gene activation, and the underlying molecular mechanisms, is therefore important for understanding the effects downstream of Notch and of signaling outputs in general. We have undertaken a comprehensive analysis of the transcriptional changes elicited within minutes of Notch activation using a short pulse of EDTA treatment. We performed a fine-scaled timecourse of the genome-wide changes in expression over 150 min after Notch activation, in parallel with an assessment of Su(H) and RNA polymerase II binding to give new insights into the Notch response. We find several different profiles of transcriptional response amongst Notch targets, which cannot be fully explained by differences in Pol II or Su(H) binding or in the arrangement of Su(H) binding sites. Our analysis demonstrates that the temporal differences arise, at least in part, from feed-forward cross-regulation between genes with different response profiles. The *E(spl)bHLH* genes appear to have a critical role in this regulation, helping to explain their pivotal contribution to the Notch response.

## Results

### Profiling the response to Notch activation

**Figure 1:**
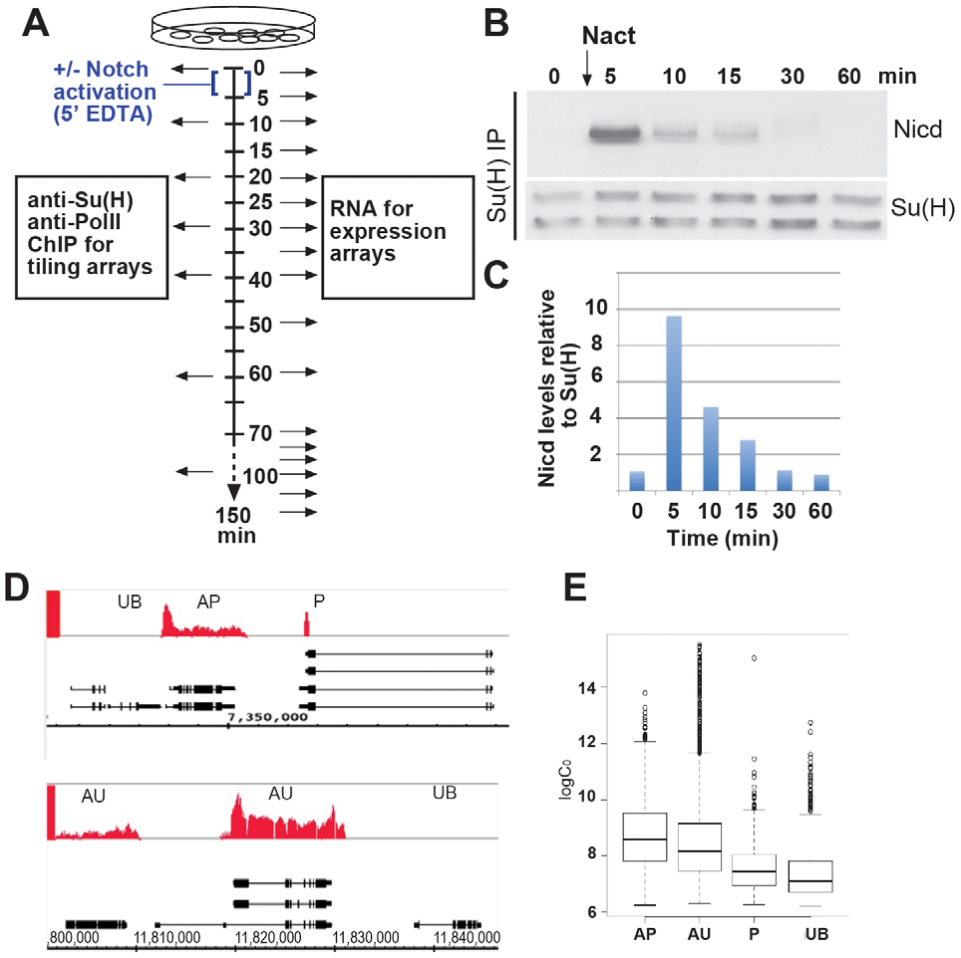
Transient activation of Notch and classification of transcripts according to Pol II class. A: Schematic outlining the experimental strategy. Arrows indicate the time-points at which data were collected. B: Levels of N^icd^ that co-immunoprecipitated with Su(H) after a 5 min pulse of Notch activation (Nact) using EDTA treatment. C: Graph shows a quantification of N^icd^ levels relative to Su(H) from B, normalised to 0 min. D: Representative genomic regions, gene models are indicated in black. Red graphs represent enrichment with anti-pSer2pSer5-Pol II relative to total input (0–0.47 fold enrichment on a log_2_ scale). Pol II binding classes, AP (active poised), P (poised), UB (unbound) and AU (active uniform) are illustrated. A ratio of log_2_(max)/log_2_(median)≥2 cut-off was used to distinguish AP from AU (see Text S1). E: Relationship between log_2_ absolute expression levels at 0 min and Pol II class at 0 min. RNA expression levels were approximated, up to a constant, by the spot intensity levels (logC_0_). The four Pol II classes have significantly different mean logC_0_ (ANOVA p value<2.2e-16). All pairs of classes, except for AU and AP, have significantly different means (pair-wise one-sided two-sample t test p values<3e-15).

To investigate whether target genes are activated with different kinetics, we used expression microarrays to profile the temporal changes in transcript levels following a pulse of Notch activation (Figure 1A). Exposing *Drosophila* DmD8 cells to a calcium chelator (EDTA) renders the Notch receptor susceptible to an activating cleavage by gamma-secretase and a concomitant release of the Notch Intracellular Domain (N^icd^) that mediates Su(H)-dependant gene activation [45]. This strategy allows precise temporal control over pathway stimulation and provides a reliable method for Notch activation [2], [29], [46], [47] although it may also elicit some non-specific effects. We found that a five min pulse of EDTA was sufficient to generate the active N^icd^ fragment which could be co-immunoprecipitated with Su(H) (Figure 1B–1C) and had largely decayed within 30 min. RNA samples collected at timed intervals following the pulse of Notch activation were used to generate labelled cDNA for hybridization to long-oligonucleotide microarrays [48]. Control non-activated samples were collected in parallel and pooled to generate a common reference, improving normalization between arrays and between the four biological replicates. Analysis of these data revealed 154 genes that showed differences in expression levels across the timecourse (Table S1). We subsequently refer to these as differentially expressed (DE) genes, which corresponded to a slightly larger number of DE transcripts (301) because more than one transcript isoform may show DE at some loci.

To correlate the induced expression changes with underlying regulatory mechanisms, the genomic binding of the active form of Pol II (Ser 2 or Ser 5 phosphorylated) and of the transcription factor Su(H) was monitored over a similar time period by chromatin immunoprecipitation (ChIP) followed by hybridisation to whole genome tiling arrays (Figure 1A). Regions of Su(H) or Pol II binding were identified by applying a peak calling algorithm that incorporated information from the magnitude of the enrichment and the number of consecutive enriched probes (see Text S1). From the Pol II binding data all transcribed regions were subdivided into four classes: Unbound (UB), no Pol II bound; Poised (P), Pol II binding at 5′ end but not within the body of the gene; active poised (AP), a peak of Pol II enrichment at the 5′ end and binding throughout the transcribed region; active uniform (AU), Pol II binding across the transcribed region (Figure 1D). Taking as an example Pol II binding prior to Notch activation, this classification of gene states at 0 min was largely consistent with the relative magnitude of the absolute levels of mRNA expression measured by our microarray analysis at the start of the time course (Figure 1E, Table S1).

### Different temporal profiles in response to Notch activation

**Figure 2:**
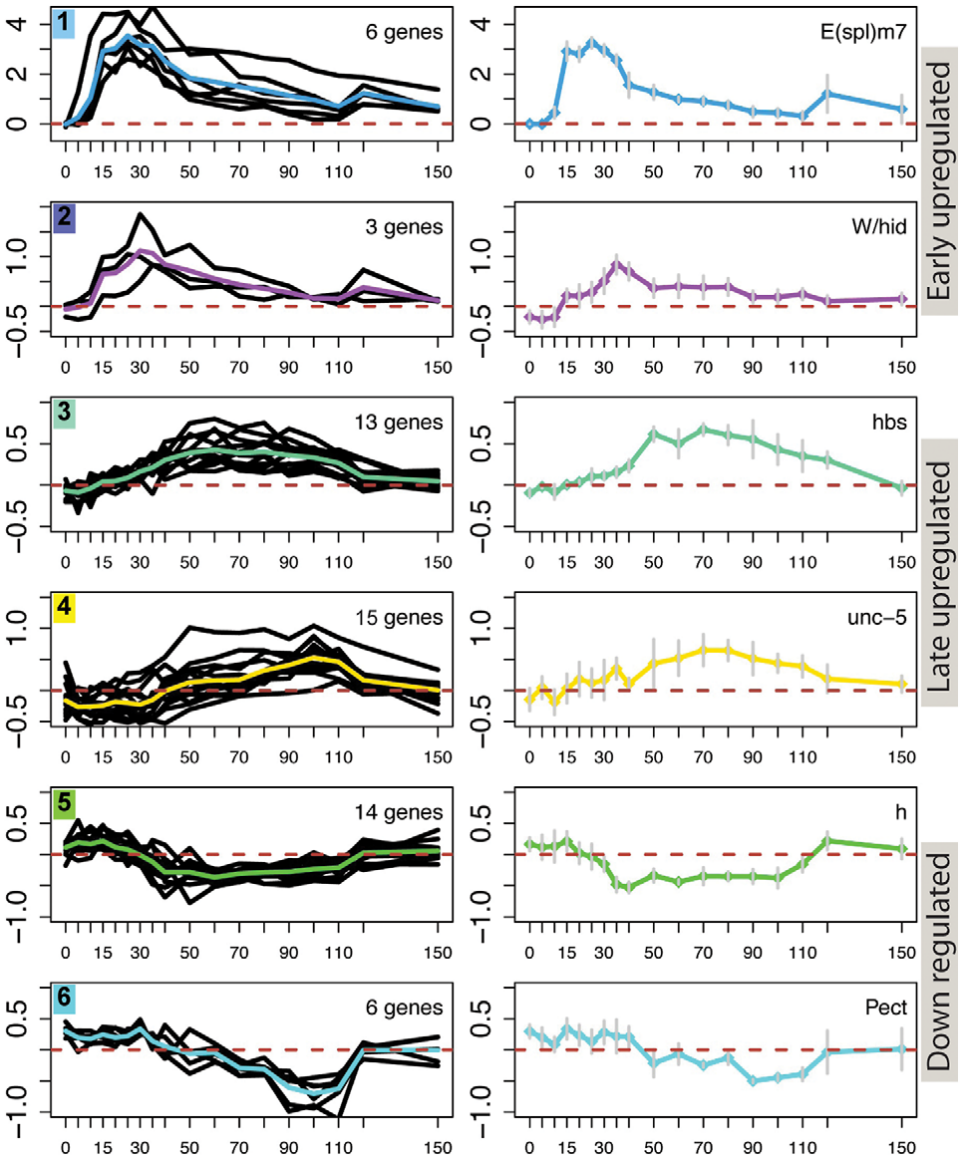
Different temporal profiles in response to Notch activation. Examples of 6 clusters of genes that exhibit different temporal expression profiles in Notch activated cells. Left graphs: profiles for all the genes in the cluster, coloured line represents the mean profile. Right graphs: profiles for a single gene from each cluster as indicated; Error bars indicate standard error of the mean from four replicates. Vertical axes for all graphs indicate median M values. Cluster types are indicated to the right of the graphs.

To determine whether the 154 DE genes could be sub-divided based on their response profiles we used an unbiased clustering analysis. This employed Bayesian clustering, with the Dirichlet-process prior, to look for and group related patterns in the data, while estimating the number of clusters directly from the data [49]. Through this analysis, we found that the RNA profiles of responsive genes could be stratified into 14 clusters, reflecting differences in response amplitude, in temporal profile and in expression “noise” (due to stochasticity in the transcription process and noise in data collection). Broadly speaking the 14 clusters were of 3 types: early upregulation (2 clusters), late upregulation (6 clusters) and down regulation (6 clusters) (Figure 2 and Figure S1). Comparing these data with our previously reported study [29], we found that all but one cluster (cluster 14) contained genes whose expression was affected by blocking N^icd^ release with a gamma-secretase inhibitor. The 98 genes allocated to the upregulated clusters were enriched (p<0.05) in functions related to response to stimulus (GO:0050896), organ development (GO:0048513) and Notch signaling (GO:0007219). Genes in the downregulated clusters were also enriched for regulation of signaling (GO:0023051; GO:0050896), notably for negative regulation (GO:0023057). For further analysis we elected to focus primarily on the six clusters that were most clearly related to the pulse of Notch activation as they showed relatively strong (>25%) up or down regulation in the first half of the time course (Figure 2).

Genes in clusters 1 and 2 had profiles indicating a rapid response to the Notch activation conditions, with maximal levels detected between 20–30 min, and were composed almost entirely of *E(spl)* genes. Of those, all but *HLHmγ* and *HLHmδ* were segregated together in cluster 1: the latter two were allocated to cluster 2 due to their smaller changes in expression levels. Cluster 2 also contained the gene *Wrinkled/hid* (*W/hid*; a regulator of apoptosis), which interestingly showed a similar response profile despite the fact that it contains three relatively large introns and is significantly larger (17 kb) than the intron-less *E(spl)* genes (<2 kb) (Figure 2). Previous studies have demonstrated that *W/hid* is a direct Notch target [29] and that Notch regulates apoptosis (reviewed in [50]). However, we did not detect any increase in activated Caspase3 in treated cells, possibly due to the transient nature of the Notch pulse (Figure S2A, S2B).

Genes in cluster 3 and cluster 4, like the remaining upregulated clusters, exhibited a more delayed response with maximal expression detected at least 60 min after induction (Figure 2; Figure S1). Cluster 3 contained genes involved in development (e.g. GO:0007444) and genes that had previously been identified as direct Notch targets [29], including *pebbled (peb), hibris* (*hbs*) and *derailed (drl)*(Figure 2). The latter makes it plausible that upregulation of at least some cluster 3 genes involves a direct input from Notch, suggesting there could be a mechanism delaying their response to the Notch pulse. Cluster 4 also contained a previously identified Notch target (*unc-5*) as well as several stress and immune response genes (e.g. *heat shock protein* 26) that could be induced under adverse conditions such as starvation (Table S1) [51], [52]. However, none were induced in previous control experiments where EDTA treatment was applied to cells that lacked Notch (data not shown). One other weakly upregulated cluster, cluster 9 also contained several developmentally related genes, including the Notch target *Egfr* [29].

Finally, clusters 5 and 6, like several other clusters, were repressed during the time course following activation (Figure 2; Figure S1). In previous experiments the downregulation of targets, such as *hairy*, was prevented by pre-treating cells with gamma-secretase inhibitor [29]. However, since N^icd^ is associated with transcriptional activation, the observed repression is more likely to be an indirect effect of Notch. Cluster 5 contained genes involved in regulating transcription and intracellular signaling cascades, including repressors such as *hairy* and *puckered*. Some of these genes exhibited an initial transient increase in expression before their RNA levels declined (Figure 2). Cluster 6 was enriched for genes involved in metabolism, a functional signature that was also found to be downregulated by Notch in other contexts [30]. Other clusters were stratified depending on the timing and magnitude of reduced expression, and included negative regulators of signaling (*SOCS36E* and *mah-jong*; cluster 12) and Notch pathway regulators (*Serrate* and *fringe*; cluster 11) (Figure S1).

### Transient and rapid recruitment of Su(H) and Pol II to *E(spl)* genes

**Figure 3:**
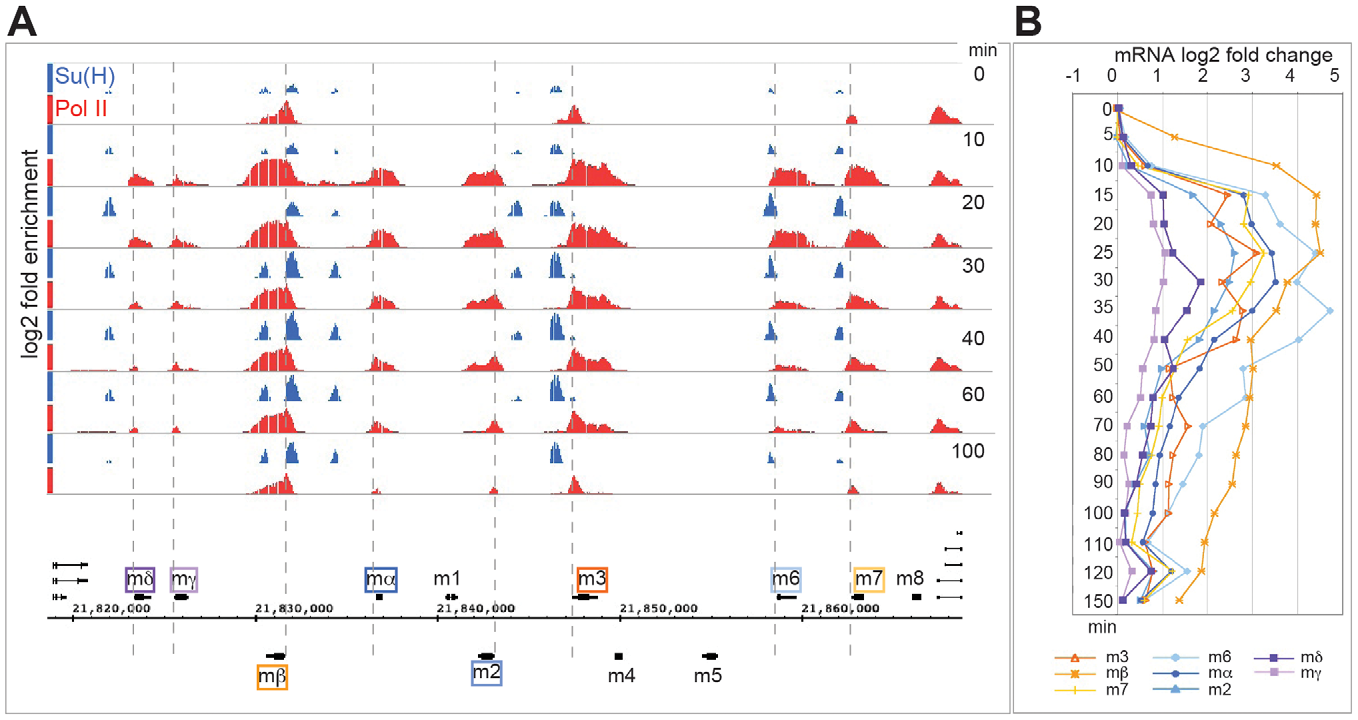
Rapid and transient recruitment of Su(H) and Pol II to genes in the *E(spl)* complex. A: Enrichment for Su(H) (blue) and Pol II (red) across the *E(spl)* complex at different time points (min) after Notch activation (Su(H) 0.5–4.5, Pol II 0–4.7 fold enrichment on a log_2_ scale). Cluster 1 genes: *mβ* (orange) *m3* (brown) and *m7* (yellow) have poised Pol II at 0 min, *m6* (light blue), *mα* (dark blue) and *m2* (mid blue) have no Pol II present at 0 min. Cluster 2 genes: *mδ* (purple) and *mγ*(pink) have no Pol II present at 0 mins. Pol II is recruited at all expressed genes by 10 mins. Su(H) occupancy increases after Notch activation at all loci. B: Log_2_ fold changes in mRNA levels for the indicated genes at different times (min) after Notch activation.

Since the response profiles from the well-characterized targets encoded within the *E(spl)* complex stratified into two different clusters, we first investigated whether Pol II and/or Su(H) binding dynamics over this genomic region could explain the differences in target gene expression (Figure 3A, 3B). For example, it has been suggested that prior recruitment of Pol II (poised Pol II) is important for rapid transcriptional responses [41]–[44]. However, this is not supported by our results. Although there is a rapid and dynamic recruitment of Pol II within 10 minutes of Notch activation, not all the rapidly responding cluster 1 genes have pre-bound Pol II. Overall, three features emerge from our data: (i) Pol II is only pre-bound at three of the eight early responding loci, and therefore does not provide a clear prediction of Notch responsive genes or rapidity of the response; (ii) recruitment of Su(H) is dynamic, showing both spatial and temporal differences in occupancy over time; (iii) Pol II intensity and Su(H) binding at T = 10 min is a good indicator of overall DE of clusters 1 & 2.

Six of the twelve genes in the *E(spl)* complex were classified into cluster 1 (*HLHm3, HLHmβ, HLHm7, m6, mα, m2*) with strong upregulation following the 5 min pulse of Notch activation, two showed weaker upregulation (*HLHmγ, HLHmδ*), segregating in cluster 2. Despite their rapid and robust upregulation, only three of the cluster 1 *E(spl)* genes had detectable Pol II present prior to activation (0 min, Figure 3A). *HLHm3* and *HLHmβ* genes exhibited strong Pol II binding close to the transcription start-site, with little or no Pol II detected across the body of the gene, suggestive of paused or poised Pol II at these loci. A low level of Pol II binding was also detected at the start of *HLHm7*. Neither *E(spl)* gene in cluster 2 had Pol II prebound. By 10 min after Notch activation, there was a rapid and robust recruitment of Pol II across all of the eight upregulated *E(spl)* genes (Figure 3A), irrespective of whether they were cluster 1 or 2, although there were much lower levels of Pol II detected at cluster 2 genes. Thus Pol II was rapidly recruited, not only to the three genes with Pol II poised at 0 min, but also to five other genes in the complex that had no pre-bound Pol II. For example, *m6* (cluster 1) had no Pol II present at 0 min, yet manifested a robust Pol II recruitment within 10 min; HLHmγ (cluster 2) also had Pol II recruited by 10 minutes although the levels were considerably lower than at other loci correlating with the lower levels of expression (Figure 3A, 3B).

Su(H) was detected at a subset (6/8) of the regulated *E(spl)* genes prior to Notch activation (Figure 3A) that corresponded to cluster 1 genes. Thus at 0 mins Su(H) binding was evident at the enhancers associated with the two poised genes, *HLHm3* and *HLHmβ* (which have previously been shown to have Su(H) bound prior to activation [47]), as well as at the enhancers associated with *HLHmα*, *HLHm2*, *HLHm6* and *HLHm7*, even though no Pol II was present at those genes, indicating that Su(H) binding is not sufficient for Pol II recruitment. Furthermore, we detected little or no Su(H) binding prior to activation at the enhancers for the cluster 2 upregulated genes, *HLHmγ* and *HLHmδ* (Figure 3A).

Strikingly the Su(H) profiles also increased after Notch activation. The enrichment increased at all the sites, reaching maximal levels at 20–30 min and declining thereafter (Figure 3A). These observations are similar to those reported previously [29], [31], [47] and suggest that Su(H) binding becomes stabilized after N activation [38], [47]. In contrast, no Su(H) binding was detected at the characterized site 5′ of *HLHmγ*, despite the fact that Pol II was recruited to the gene and that the region was shown to provide Su(H) dependent regulation to reporter genes [39], [53]. We also note that neither Su(H) nor Pol II were recruited to the *m4* - *HLHm5* gene region at any time, in agreement with their lack of activity in DmD8 cells, although both genes are Notch responsive in other contexts [36], [54].

### Relationship of Pol II class and Su(H) binding to response profiles

**Figure 4:**
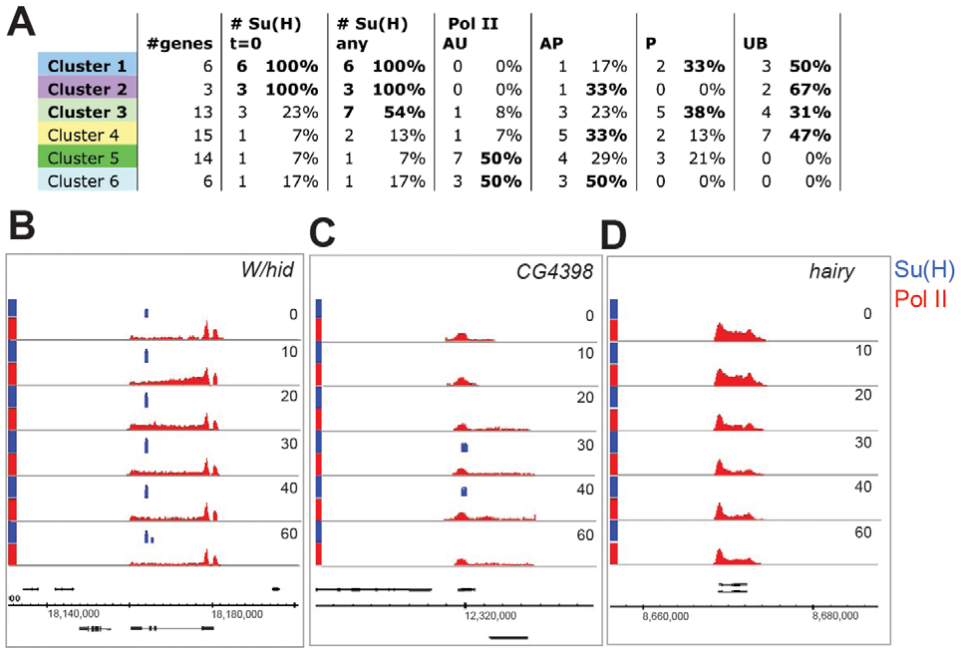
Patterns of Su(H) and Pol II recruitment. A: Table showing, for each cluster, the proportion of genes with Su(H) binding within 10 kb [ # Su(H)] and with each Pol II state (Pol II: AU, AP, P and UB as described in main text). In cases where individual transcripts of a gene had different Pol II states, the gene was assigned a state as follows: AU>AP>P>UB. Conditions where >30% of genes are ascribed to a particular class are indicated in bold. B–D: Enrichment for Su(H) (blue) and Pol II (red) across the *W/hid* (B), *CG4398* (C) and *hairy* (D) genes at different time points (min) after Notch activation (Su(H) 0.5–4.5, Pol II 0–4.7 fold enrichment on a log_2_ scale).

Extending the analysis to the other temporal clusters, we considered their relationship to Pol II class at 0 min and to Su(H) binding (Figure 4A, Tables S1 and S2) to determine whether the clusters exhibited different patterns of recruitment. Together the analysis suggests that Pol II class and Su(H) binding influence the response profile, although there is no strict relationship between either parameter and the response cluster.

As discussed above, the strongest responding groups (1 and 2) consist primarily of genes from the *E(spl)* complex where the majority of genes recruit Pol II de novo (i.e. they change from unbound, UB, to active, AP/AU, after activation) and hence were enriched for the UB class at 0 min. We note that the other member of cluster 2, *W/hid*, was P class at 0 mins (Figure 4A, 4B) and, like the cluster 1 genes, was associated with Su(H) binding at 0 mins. Confirming the role of Su(H) in *W/hid* regulation, we found that mutation of the Su(H) motifs underlying the peak region abolished Notch responsiveness in transient transfection assays (Figure S2C). The other cluster 2 genes, *HLHmγ* and *HLHmδ*, were also associated with 0 min peaks of Su(H) binding within 10 kb, although there was only a single site of Su(H) binding that was located in the region 5′ of HLHmδ and that showed increased binding after activation (Figure 3A, Figure 4A and 4B).

**Figure 5:**
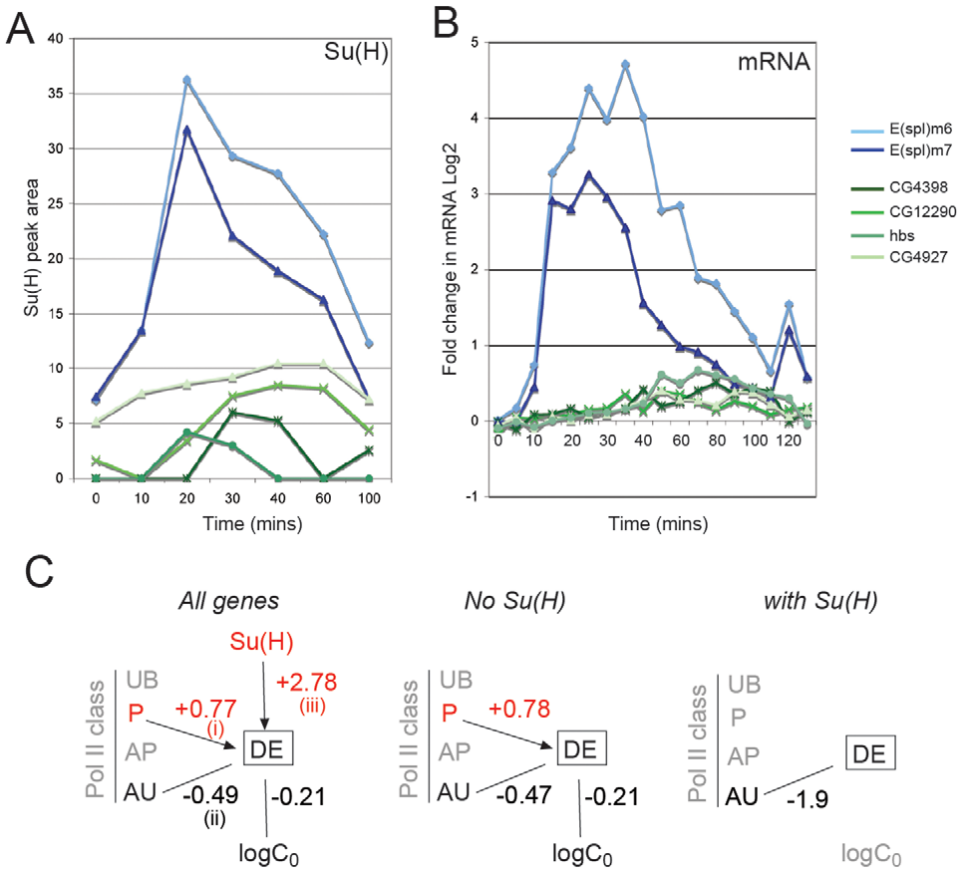
Relationship between Su(H) binding and gene responses. A: Enrichment for Su(H) at the indicated times, calculated from the area under the peak of ChIP enrichment (Blue: genes from cluster 1; Green: genes from cluster 3). B: Log_2_ fold changes in mRNA levels for the corresponding genes. C: Diagram illustrating whether Su(H) binding, Pol II class and initial absolute expression level (log C_0_) have statistically significant positive (red) or negative (black) effects on the log odds of differential expression (DE). Effects were calculated using logistic regression models (see Text S1). Solid lines indicate statistically significant effects (p<0.005 after adjusting for multiple testing); the numbers indicate the estimated mean effect on the log odds (see Text S1). For example, in considering all expressed genes these effects equate to: (i) Su(H) bound within 10 kb at 0 min increases odds of DE by e^2.78^ = 16.12. (ii) P class increases the odds of DE by e^0.77^ = 2.16, (iii) AU class decreases odds of DE by e^−0.49^ = 0.61.

Genes in clusters 3 and 4, which showed moderate or weak changes in expression, were distributed between the UB, P, and AP Pol II classes at 0 mins (Figure 4A), and most showed increased Pol II recruitment by 20–30 mins (e.g. Figure 4C). Thus, although 40–50% of loci had some poised polymerase, a substantial fraction of the upregulated genes in these clusters, like the cluster 1 genes, were not bound by polymerase prior to activation (UB at 0 min). Of the two clusters, only cluster 3 was strongly enriched for genes located in proximity to Su(H) peaks (Figure 4A; Tables S1, S2). At those loci the Su(H) binding profiles, as determined by peak area, largely mirrored the expression profiles. The levels of Su(H) binding in cluster 3 genes thus equated with the upregulation of mRNA, although they were considerably reduced in magnitude compared with the sites associated with cluster 1 genes (Figure 5A, 5B).

Finally, clusters with repressed profiles were enriched in AU and AP class at 0 min compared to the other clusters, and many had a detectable decrease in Pol II recruitment by 20 min after activation (e.g. *hairy*, Figure 4D). Since the observed repression is likely to be an indirect effect of Notch, we did not expect to find any association with Su(H) binding at these genes. Surprisingly however, a small number of genes were associated with Su(H) peaks (Table S1). These also showed a brief increase in expression, which correlated with an increase in Pol II recruitment (e.g. *edl*, *argos*, Figure S3), before the mRNA and Pol II levels declined, suggesting that they could receive a transient input from N^icd^.

Together the analysis suggests that the different response clusters have a bias towards different Pol II classes and Su(H) binding profiles. Thus, although calculations indicate that, overall, P class (poised polymerase) transcripts had an increased likelihood of DE whereas the likelihood for active uniform (AU) class was significantly reduced (Figure 5C; see Text S1), the distribution of Pol II classes differed according to the response profile. Likewise, upregulated DE genes were more likely than other genes to have Su(H) binding within 10 kb at some point during the timecourse (Figure 5C) and to attain a higher level of peak expression than other DE genes over the timecourse, however the timing and magnitude of recruitment, based on peak area, differed (Figure 5A).

### Relationship of Su(H) motifs and occupancy to DE

One possible explanation for the differences in Su(H) recruitment is that the target enhancers differ in the number, arrangement or affinity of Su(H) binding sites. However, we found no differences in the number or apparent affinity of Su(H) motifs underlying the Su(H) binding peaks associated with genes in different clusters, with each peak containing one or more high affinity sites, based on matches to previously identified Su(H) binding motifs (Table S3) [29]. In contrast, we found that all of the peaks associated with *E(spl)* genes in clusters 1 and 2 contained at least one paired Su(H) site (SPS). However, no SPS motif was present in the *W/hid* associated peak although this gene responded with a similar profile to the *E(spl)* genes in the same cluster (Figure 2). The only other peak containing an SPS motif was associated with *corn*, which was not significantly upregulated after the Notch pulse.

Furthermore, although Su(H) binding increased the likelihood of differential expression (Figure 5C) only 26% of the positions where Su(H) was bound at 0 min were within 10 kb of a gene that exhibited DE over the timecourse (Table S2). This conclusion was not altered when the analysis was extended to the nearest 2 neighbouring genes on all strands, irrespective of distance. For selected examples of genes with Su(H) binding that were not DE we can be confident that the P or AP class neighbouring genes were potentially Notch regulated as they have been identified as targets in other experiments (e.g. *Notch, corn*). However, it is possible that some other sites, where we cannot reliably associate them with neighbouring DE genes, may be acting over a much longer range to confer a Notch response. Nevertheless, despite the fact that Su(H) occupancy predisposed genes to DE (Figure 5C), many sites of Su(H) binding did not elicit a detectable response to the short pulse of Notch activation from any neighbouring genes.

### Feed-forward interactions shape the response

The response profiles could be shaped by many factors including differences in mRNA stability as well as in transcriptional initiation. Since we observed that the Su(H) binding and Pol II recruitment were related to the response, it is likely that at least some of the effects were due to differences in the timing of transcription (e.g. for cluster 3 genes). One model to account for delayed upregulation is a feed-forward model in which early expressed genes contribute to the regulation of the later ones. For example, an early responding gene might encode a transcription factor needed to upregulate the late responding targets. The fact that the early acting genes consisted of *W/hid*, which has no known transcription factor activity and members of the *E(spl)* complex, which encode powerful bHLH repressors, makes this simple scenario unlikely. Furthermore, genes from several late clusters (e.g. *hbs, dpn, Egfr)* were still upregulated in cycloheximide treated cells, demonstrating that their upregulation was not dependent on de novo synthesis of a trans-acting factor (Figure S4).

**Figure 6:**
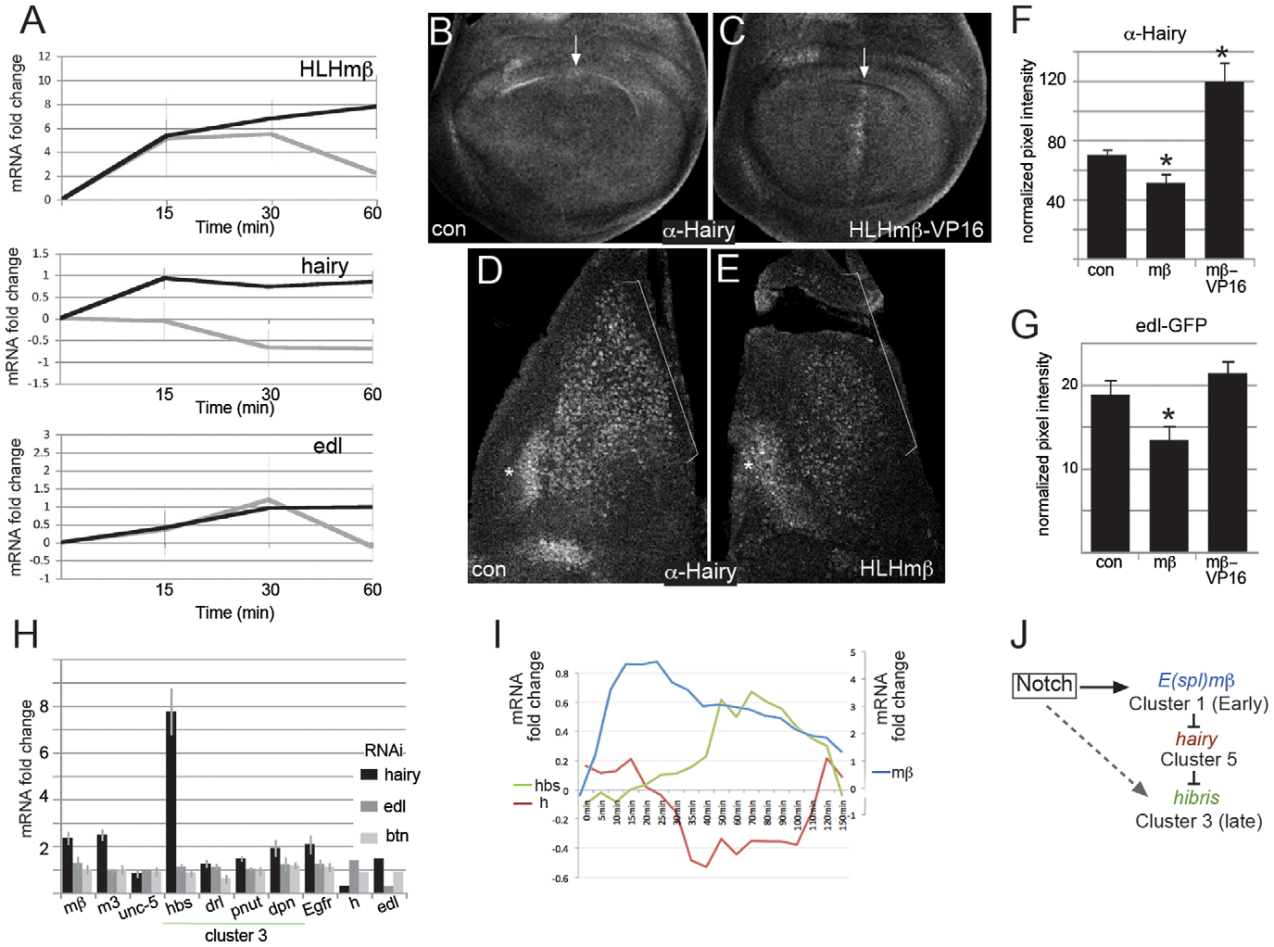
Evidence for cross-regulatory relationships between genes in different clusters. A: mRNA expression levels of the indicated genes in Notch activated cells relative to controls (log_2_) in untreated (grey) and cycloheximide treated (black) cells at the times indicated. Cells were exposed to cycloheximide for 60 min prior to Notch activation at 0 min as well as during the timecourse. B,C: Expression of Hairy in the wing imaginal disc pouch from control (B, *ptc-Gal4 ; UAS-lacZ*) and HLHmβ-VP16 (C, *ptc-Gal4* ; *UAS-HLHmβ-VP16*), arrows indicate the stripe of HLHmβ-VP16 expression where Hairy is induced. D,E: Expression of Hairy in muscle progenitors (brackets) from control wing imaginal discs (D, *1151-Gal4*; *UAS-lacZ*) and from those expressing HLHmβ (E, *1151-Gal4*; *UAS-HLHmβ*). Hairy is reduced in the muscle progenitors (brackets) but not in neighbouring epithelial cells (asterisks). F,G: Quantification of expression levels of Hairy (F) and *edl-GFP* (G) in muscle progenitors expressing β-galactosidase (con), HLHmβ (mβ) or HLHmβ-VP16 (mβ-VP16). Average pixel intensities from a defined region within the expression domain were measured using ImageJ and normalized relative to background levels from a comparable region in the same discs, >5 discs per genotype. Error bars indicate standard error of the mean. Asterisks indicate that results are significantly different from control (p≤0.05; using an unpaired, 2-tailed student T-test). H: Fold change of the indicated mRNAs in cells treated with dsRNA against *hairy*, *edl* or *btn* in comparison to controls (no RNAi). RNA levels were reduced by 65%, 71% and 61% for *hairy*, *edl* and *btn* respectively. These experiments were performed in the basal state (no Notch activation). Bars represent the average of three biological replicates and error bars indicate standard error of the mean. I: Log_2_ fold changes in mRNA levels of *hairy* (*h*, brown) *hibris* (*hbs*, green) and *HLHm*β (*m*β, blue) from the microarray study. Scale for *hbs* and *h* is indicated by left axis and for *m*β, which had larger fold changes, by right axis. J: Summary model of the feed-forward regulatory relationships, dotted line indicates that the direct regulation of *hbs* (cluster 3) by Notch signaling has not been directly tested here, although *hbs* and other genes in cluster 3 exhibit Su(H) binding, which implies that at least some undergo direct Notch regulation.

Prompted to consider alternative regulatory interactions, we realized that the profiles might fit an alternative feed-forward model, where the early acting genes downregulate a repressor so enabling expression of the late upregulated genes. Clusters 5 and 6, which included several negative regulators, were downregulated shortly after cluster 1 bHLH repressors had reached their peak, making them plausible targets of the early *E(spl)bHLH* gene products (Figure 2). Since the multiple bHLH repressors in cluster 1 are known to function redundantly [55], [56], it would require knock down of multiple genes simultaneously to test functional relationships in cultured cells by RNAi. We therefore first asked whether the repression of two cluster 5 genes (*hairy* and *edl*) required de novo protein synthesis by treating the cells with cycloheximide (CHX). (Figure 6A). Under these conditions, *HLHmβ* mRNA showed similar levels of induction to untreated controls although, interestingly, the expression levels no longer declined after 30 minutes. This suggests that the decrease in *HLHmβ* expression is a consequence of de novo protein synthesis and fits with the observations that the bHLH repressors can feed back on their own expression (contributing to the cyclic expression of *HES* genes that has been observed in a variety of precursor cell types e.g. [57]–[59]). The effects of cycloheximide on *hairy* mRNA were also striking, *hairy* was no longer repressed following Notch activation, levels increased within 15 minutes and remained high throughout the time course. Likewise, the profile of *edl* expression also changed to one of continued upregulation in the presence of cycloheximide. Thus *hairy* and *edl* repression were dependent on de novo protein synthesis, consistent with the hypothesis that this is due to inhibition mediated by the early induced *E(spl)bHLH* proteins.

We next turned to an *in vivo* assay, taking advantage of flies carrying an inducible *HLHmβ* construct, and monitored the consequences of elevated *HLHmβ* expression on the cluster 5 gene *hairy* (Figure 6B–6F). Normally, *hairy* is expressed in the muscle progenitors (*in vivo* correlates of the DmD8 cells; bracket, Figure 6D, 6E) at levels that are similar to neighbouring epithelial cell domain (asterisk, Figure 6D, 6E). Expression of *HLHmβ* in muscle progenitors inhibited *hairy* expression; Hairy levels were clearly reduced compared to the neighbouring domain (Figure 6E, 6F). Conversely, expression of *HLHmβ*-VP16, in which the terminal *HLHmβ* WRPW repressor recruiting motif was replaced with a viral VP16 activation domain, either in the muscle progenitors or in a stripe in the wing imaginal disc, was sufficient to promote ectopic Hairy expression (Figure 6B, 6C, 6F). Furthermore, *HLHmβ* also reduced expression of an *edl* reporter (*edl-GFP*) although the effects of *HLHmβ*-VP16 were not significant in this assay (Figure 6G). Together these results support the proposed regulatory relationship between E(spl)bHLH and the cluster 5 gene *hairy* and suggest the model may extend to other cluster 5 genes such as *edl*.

The fact that *hairy* and several other cluster 5 genes themselves encode potential repressors raised the possibility that one or more of these repressors could contribute to the delayed expression of the late responding genes. Downregulation of such a repressor could be the critical step that permits the expression of the late responding clusters. We therefore tested whether RNAi knock down of *hairy*, *edl* or *btn* was sufficient to cause upregulation of selected late expressed genes (Figure 6H). The knock down of *hairy* led to a significant increase in three of the four cluster 3 genes tested (*hbs, pnut, dpn*) with *hbs* expression showing the greatest fold-change. It is possible that *drl* and some other genes failed to be upregulated after *hairy* knock-down, because they additionally required Notch-mediated activation, a possibility that is supported by Su(H) binding within the intron of *drl* at the 20 min time-point. However, it was not technically feasible to combine RNAi treatment with the Notch activation protocol. No expression changes were detected after knockdown of *edl* or *btn* (Figure 6H) suggesting that *hairy* is a more likely candidate for mediating cross-regulation of late upregulated targets in cluster 3 (Figure 6I, 6J). In support of this hypothesis, 77% of genes from cluster 3 (10/13) have one or more matches to Hairy consensus binding motifs within 5 kb of the transcription unit in comparison with 47% of cluster 4 genes (7/15)) although this difference is no longer evident when the analysis is extended to a 10 kb region around the genes (85% of cluster 3 genes and 80% of cluster 4 genes) (Table S3).

### Sensitivity to the dose of active Notch

**Figure 7:**
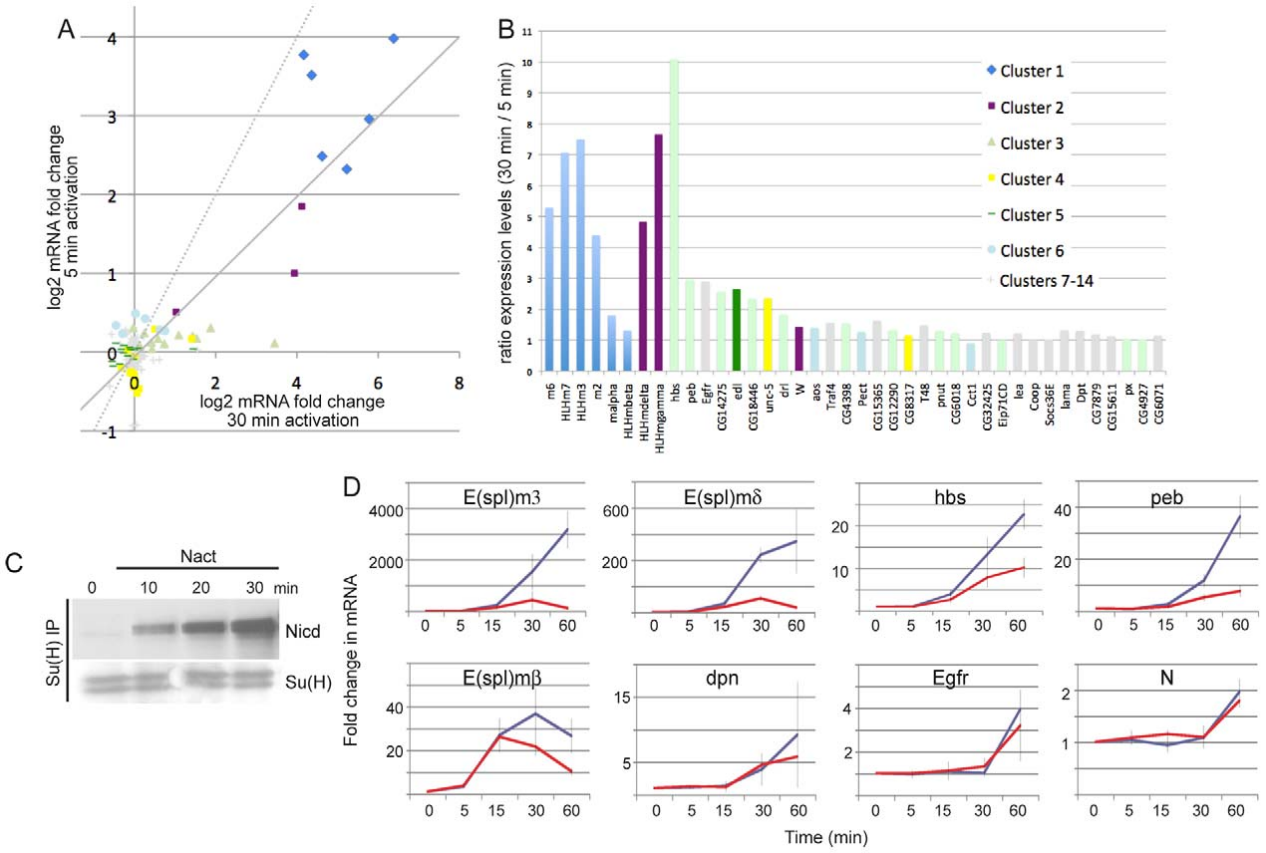
Differential sensitivities to the dose of Notch activation. A: Comparison of fold changes in mRNA expression levels (log_2_) from 5 and 30 min Notch activation regimes. Symbols indicate fold-change at 30 min time-point after commencing activation (EDTA) treatment, where colours represent cluster assignments according to the legend in B. Dashed line represents the expected trend if each treatment produced the same response; solid line indicates the line of best fit from the data (regression coefficient = 0.84, r2 = 0.71). B: Ratio of the fold change in mRNA levels at 30 min, with a 30 versus 5 min treatment for the indicated genes. Higher bars indicate greater sensitivity to the differences in the activation regime. Colours indicate cluster assignments as in the legend. C: Levels of N^icd^ that co-immunoprecipitate with Su(H) under continuous treatment for the times indicated (compare with Figure 1A). D: Fold changes for the indicated mRNAs at the times indicated (red lines: 5 min activation, blue lines: continuous activation). T = 0 corresponds to the time at which the activation regime commenced. Error bars indicate standard error of the mean from four biological replicates.

Another contributory factor to the different gene responses could be their sensitivity to Notch signal duration and/or intensity. Differences in response threshold might also explain the observation that the majority (74%) of Su(H) binding regions were not associated with neighbouring genes that were differentially expressed. To examine this question in more detail we first compared the response of the 154 DE genes to a 5 min pulse of Notch activation with our previously described results obtained after 30 min of Notch activation (Figure 7A) [29]. This demonstrated that, although the shorter pulse in general elicited a weaker response, most genes had quite similar responses under the two conditions (correlation coefficient 0.84). This was further evident when the ratios of responses were plotted as the majority of targets showed less than 2-fold difference between the short and long Notch induction (Figure 7B). However, 13 genes exhibited >2 fold difference in response. These were predominantly genes in clusters 1–3, i.e. with the strongest response following the 5 min stimulation, but not all the genes in those clusters were similarly affected (5/7 genes from cluster 1, 2/3 from cluster 2 and 4/13 genes from cluster 3). Of the repressed genes, only one (*edl*) showed >2 fold difference in response although many, such as *hairy*, were repressed under both conditions. From this analysis it appears that in general the different signaling regimes produce a similar response and that although some genes have greater sensitivity to signal duration/levels this is not sufficient to explain the different response profiles. Although there was no functional enrichment amongst the genes with greater dose sensitivity, we note that two are regulators of Ras signaling, possibly reflecting the importance of fine-tuned cross-talk between Notch and Ras pathways.

To investigate the effects of signal strength on the timing of response profiles we directly compared expression of several genes in response to a 5 min pulse or continuous stimulation (Figure 7C, 7D). Prolonged stimulation resulted in longer duration and higher levels of N^icd^ (Figure 7C). Some genes showed stronger induction in continuous activation conditions (e.g. *HLHm3*, *HLHmδ*, *hbs*, *peb*) suggesting that their transcription is sensitive to the levels of N^icd^. Others showed little difference in expression levels (*HLHmβ*, *dpn*, *Egfr*) suggesting that N^icd^ is permissive rather than instructive at these loci. Notably, however, the temporal profiles were similar under the two conditions, even for those genes that were more strongly induced (Figure 7D). Thus the increase in signaling from the more extended treatment was not accompanied by an earlier onset of upregulation.

Seven genes that were associated with Su(H) binding and that had Pol II enriched at their 5′ regions were not detected as significantly DE after 5 min of stimulation, although they were after 30 minutes. One example is *Notch*, which exhibited robust Su(H) binding and was classified as AP at 0 min. However, when we compared the expression profiles of *Notch* mRNA with the 5 min pulse and continuous stimulation, there was little difference in the response (Figure 7D). Notably, the fold-change in expression was small (2-fold) in both cases. This may explain the failure to detect significant upregulation in the array experiments after 5 min activation if there was more variability in the response with the short stimulus. For example, genes that are already transcribed and undergo small changes in expression levels or genes that have variable/stochastic responses (e.g. more cell to cell variability) may not fulfil the test for significance between replicates. Other loci associated with Su(H) binding may therefore have similar characteristics to *Notch*, requiring prolonged stimulation before they achieve consistent expression. Nevertheless, these results suggest that Su(H) and Pol II binding are not sufficient to generate a reproducible response to the short stimulation.

## Discussion

Performing a fine-scaled temporal analysis of the transcriptional response to Notch has revealed that the expression profiles of responding genes stratify into several classes. These differed in the timing, levels and direction of the expression changes. For example, the upregulated genes subdivided into clusters with rapid responses (within 10–15 min) and more delayed responses (>30 min). The different profiles could be explained, at least in part, through regulatory interactions between the different targets. Notably, we found evidence for feed-forward repression by the early responding *E(spl)bHLH* genes and we propose that this generates a temporal window for the delayed upregulation of later responding genes, by decreasing the expression of the repressor *hairy*. A pivotal role in the regulation of late responding targets could reconcile the observed critical functions of *E(spl)bHLH/Hes* genes in the Notch response [18], [25]–[28] with the large numbers of direct targets that have been identified in recent genome-wide studies [29]–[32]. This illustrates how the temporal analysis of signaling outputs can be informative for unravelling the underlying regulatory networks.

In the cells analyzed, *E(spl)* complex genes were almost unique in the rapidity and magnitude of their response to the 5 min pulse of Notch activity. We note however that not all of the rapidly responding genes had Pol II present at the promoter prior to Notch activation, suggesting that N^icd^ must be able to efficiently recruit Pol II de novo. Only one other gene, the pro-apoptotic factor *W/hid*, stratified into the early responding clusters. *W/hid* differs from *E(spl)* genes in possessing introns and requiring splicing, which has previously been suggested as an important factor in regulating the timing of transcriptional responses [60]. *W/hid* also lacks the paired SPS Su(H) binding motif that is a feature of the *Hes* gene targets and that could contribute to their efficiency of activation by promoting N^icd^/Su(H) dimerization [35]–[40]. Nevertheless the *W/hid* profile of upregulation was very similar to that of two *E(spl)* genes, indicating that neither lack of introns, nor the presence of an SPS motif, nor location within the *E(spl)* complex were pre-requisites for rapid upregulation.

Other upregulated targets exhibited a more delayed response. This raises the question of what mechanisms were responsible for the delay. One contributory factor to response profiles could be mRNA stability, clusters with more stable mRNAs would be predicted to have a lag in their response compared to clusters with less stable mRNAs [61]–[63]. Although this may have an influence, it cannot fully explain the observations that Pol II recruitment was delayed at several of the late responding genes (e.g. *hbs* and *CG4398*) compared to the rapidly responding classes and in general there was a shift in the onset as well as the peak of the profiles. Neither of these features would be expected if profile differences were due to mRNA stability alone and suggest that a delay in transcriptional activation is a contributory factor. We propose that one reason for the delay in transcriptional activity of late responding genes is that Notch activation has to first combat the presence of repressor(s) at those loci. In this model, early responding *E(spl)bHLH* genes inhibit the expression of Hairy, and possibly other existing repressor(s), thereby releasing the late responding genes from repression and enabling their response to Notch (Figure 6). Our results from testing the cross-regulatory relationships support this model for at least some targets. It will be interesting to determine whether such a model is more generally relevant and extends to other signalling pathways.

As the proposed mechanism involves alleviation of repression, the later responding targets need not, a priori, be direct targets of Notch activity. Thus, these loci may not receive direct input from Notch but are “released” by the drop in Hairy (or other repressor) levels for upregulation by other transcription factors. We consider this a likely model for the delayed upregulation of cluster 4 genes which are largely without Su(H) binding. However, the associated Su(H) binding and previous analyses indicate that some cluster 3 late responding genes (e.g. *hbs, peb/hnt*) could also require a direct input from Notch in addition to the release from repression. The profile of Su(H) binding at cluster 3 targets was consistent with this possibility, as there was less Su(H) present and often the binding was delayed until 20 or 30 min after activation, despite the fact that most contained multiple matches to high affinity Su(H) motifs. We note also that 3 additional cluster 3 genes exhibited Su(H) binding after stronger Notch inductions [29] and that not all targets could be upregulated by *hairy* knockdown alone. For these reasons we consider it plausible that cluster 3 genes require input from Notch directly for their upregulation and that the decay of N^icd^ after 30 minutes could be one explanation for their low level of upregulation.

The model that feed-forward repression is involved in shaping the response to Notch has several implications. First, it could explain why mutations in *E(spl)/Hes* can phenocopy Notch knockdown [18], [25]–[28], despite the fact that recent studies have identified a large spectrum of other direct Notch targets [24], [29], [31]–[33], because the model implies that *E(spl)* genes are the primary responders with a pivotal role in permitting the expression of other targets. Second, it could account for situations where Notch activity appears to enable subsequent changes in cell fates through repression of a Hairy family gene [64], [65]. Third, this model suggests that there is an underlying buffer, in the form of Hairy (and possibly other repressors), which could prevent many targets from responding to very low levels of Notch. Although Hairy is a well-characterized transcriptional repressor during segmentation, the concept that it can function as a gate-keeper for Notch response is novel. Here we propose that *hairy* repression by the early targets of Notch activity could feed forward to enable the expression of other Notch responsive genes. Other signals/factors might also be able to exert an impact on Notch activity via their effects on *hairy* regulation.

Several targets were unaffected by *hairy* knock down suggesting there could be other repressors that perform a similar function to Hairy, or that there are other mechanisms involved in attenuating the response of late upregulated targets. The existence of other repressors could also explain why only a relatively small proportion of Su(H) bound regions (26%) were associated with differentially expressed genes and would be consistent with previously proposed models in which Su(H) binding alone is insufficient for target gene regulation [53]. Thus, the combination of Su(H) binding and poised polymerase was not necessarily sufficient to guarantee a response to Notch activity from neighbouring genes, despite the fact that Su(H) binding increased after Notch activation (e.g. at *Notch* itself) as it did at genes that exhibited DE. Non-responding targets may be prevented from N^icd^ induced upregulation by repressors that are not downregulated by E(spl)bHLH, or by the lack of a co-operating activator. If the latter, it would imply that these genes have a requirement for additional activators even after the recruitment of Su(H). An alternative explanation is that higher levels of N^icd^ are required. Dose dependent differences in the Notch response have been seen in mammary epithelial cells [66] and in umbilical cord stem cells [67]. However, a comparison with longer periods of activation demonstrated that, although some genes differed in their sensitivity to the “dose” of Notch activity, the majority of loci exhibited similar upregulation under both regimes. Signal duration was therefore not sufficient to explain why some genes responded poorly, although it is possible that even longer signal durations are required to induce a response. Cell-type differences in gene sensitivity may thus be shaped by the presence of specific repressors. Analysis of the gene responses to different activation regimes in a range of cell-types will be needed to distinguish these possibilities.

## Materials and Methods

### Cell culture and Notch activation regimes

DmD8 cells were cultured in Schneider's medium (Invitrogen) supplemented with 10% FBS (Sigma), 5 µg/ml insulin (Sigma) and 5% penicillin/streptomycin (Sigma) according to standard protocols. Notch signaling was initiated by replacing cell media with 2 mM EDTA in PBS. For the majority of experiments cells were stimulated for 5 minutes before washing out EDTA using normal culture media. For continuous activation experiments, cells were incubated in EDTA for the times indicated. For control samples, media was replaced with fresh media to mimic the addition and removal of EDTA in corresponding experimental samples.

Quantification of mRNA was performed using standard qPCR techniques. RNA was purified as described in Text S1 and reverse transcribed using MMLV-Reverse transcriptase (Promega) primed with random hexamer oligonucleotides (Promega) to produce cDNA. Relative cDNA concentrations were quantified using QuantiTec SYBR Green PCR mix (Qiagen) according to manufacturers instructions. PCR reactions were performed using a LightCycler 480 (Roche) quantitative PCR machine. Primers are described in Table S4.

To monitor N^icd^ in transcription complexes, its association with Su(H) was determined by co-immunoprecipitation, performed using standard techniques. Briefly, 2 µg Su(H) antibody (Santa Cruz Biotechnology, sc-15813) was incubated with protein G agarose beads at 4°C for 4 hours. Beads were then incubated overnight at 4°C with lysate from Dmd8 cells treated with EDTA for the times indicated. Samples were washed twice before resuspending in SDS loading buffer. Western blots were performed using standard techniques and were visualised using ECL reagents (GE Healthcare). Antibodies used were goat α-Su(H) (1/200) (Santa Cruz Biotechnology) and mouse α-N^icd^ (1/100) (Developmental Studies Hybridoma Bank).

### RNA profiling using expression microarrays

For each sample (18 time points, 4 replicate experiments), RNA was purified from cells and the poly-A tailed mRNA was reverse transcribed using oligo(dT)23 primers in the presence of Cy3- or Cy5-dCTP to generate probes for hybridization to long oligonucleotides microarrays (Flychip FL003; GEO platform accession GPL8244) as described in Text S1. Intensity values for each probe were extracted from scanned arrays using Dapple [68]. The R package *limma* [69] was then used to normalize the arrays. Significant differential expression over the time course was then detected using EDGE software [70]. See Table S5 for resulting expression data and Text S1 for further details of normalization, quality control and statistical analysis. Results have been deposited in Gene Expression Omnibus (GEO Series record GSE35557 http://www.ncbi.nlm.nih.gov/geo/). The method used to perform the gene clustering is available as an R-package: http://cran.r-project.org/web/packages/DIRECT/index.html

### Chromatin immunoprecipitation and hybridization to genomic tiling arrays

ChIP: Further details are provided in Text S1. In brief, for each sample (7 time points, 3 replicates) cross-linked chromatin was fragmented by sonication to an average length of approximately 500 bp and precleared by addition of rabbit IgG and protein G agarose beads (Santa Cruz Biotechnology) before incubation at 4°C overnight with protein G agarose beads that had been pre-incubated with 2 µg Su(H) antibody (Santa Cruz Biotechnology) or 2 µg Pol II antibody (abcam). After washing, chromatin was eluted from the agarose beads with elution buffer (100 mM NaHCO_3_, 1% SDS) for 10 minutes with vigorous shaking before reversing the cross-links by incubation at 65°C for 5 hours in NaCl (0.27 M final concentration). Remaining proteins were removed by incubating with proteinase-K at 55°C overnight. DNA was then purified by phenol/chloroform extraction and ethanol precipitation. For array analysis, DNA fragments and total input samples were amplified by ligation mediated PCR and labelled for hybridisation to NimbleGen *D. melanogaster* 2.1 M Whole-Genome Tiling Arrays in the NimbelGen hybridisation station at 42°C (mix mode B) for 18 hours. Post-hybridisation washes were performed according to the NimbleGen Wash Buffer Kit instructions. Intensity values for each probe were extracted using the NimbleScan software. For each antibody, results were obtained for 3 replicates at 7 time points (21 arrays). Details of normalization and data analysis are in Text S1. Results are included in GEO series GSE35557.

### *In vivo* assays

Fly stocks: *In vivo* analyses were performed using the following fly lines: *1151-Gal4* [71], *ptc-Gal4* [72], *UAS-HLHmβ* [25] and *UAS-HLHmβ-VP16* [73]. All crosses were performed at 25°C using standard conditions.

Immunostaining: Immunofluorescence was performed as described previously [74], and expression of proteins/reporters in imaginal discs from third instar larvae was analyzed as described previously [29]. Antibodies used were mouse α-Hairy (1/100; A gift from S. Pinchin and D. Ish-Horowicz), α-GFP (1/1000; Molecular Probes), α-cleaved Caspase-3 (1/1000; Cell Signaling Technology) and fluorophore conjugated secondary antibodies (Jackson ImmunoResearch). To quantify expression levels, average pixel intensities from the manipulated territory were measured using ImageJ and normalized relative to background levels in the same discs, >5 discs were quantified per genotype.

### Prediction of Su(H) and Hairy binding sites

Alignment matrices for Su(H) [29] and Hairy [75] were built based on a compilation of previously published binding sites. The motif scanner nmscan from the NestedMICA package [76] was used for genome-wide motif matching. SPS arrangements were determined as combinations of two sites in opposite direction spaced by 10 to 22 nucleotides.

## Supporting Information

Figure S1 Clustered expression profiles of DE genes. Graphs show log_2_ fold change in mRNA levels over time (min) for gene clusters. Black lines represent profiles of individual genes and coloured lines show the mean response of the cluster.
(TIF)

Figure S2 Activated Caspase 3 in treated and untreated cells and role of Su(H) motifs in *W/hid* enhancer. A. Images show staining for activated Caspase 3 (red) in DmD8 cells at indicated times following a 5 minute pulse of EDTA treatment (Nact/EDTA) or control treatment. The turquoise channel shows a phase contrast image of the field. B. Average number of cells containing activated Caspase 3 per field, quantified from a minimum of 5 fields per condition. Error bars indicate standard error of the mean. No significant differences were found between Notch activated and control conditions (30 min – p = 0.34, 60 min – p = 0.79). C. Response of the indicated enhancers to N^icd^ in transient transfection assays in *Drosophila* cells, expressed as fold-change (dark bars) relative to expression levels in the absence of N^icd^ (pale bars). Mutating Su(H) motifs in the *W/hid* enhancer (green bars) abolishes responsiveness to N^icd^. Error bars indicate standard error of the mean from 3 biological replicates. *E(spl)m3*, *control* and un-mutated *W/hid* luciferase reporters were described previously [29]. Su(H) sites in the *W/hid* enhancer were mutated using oligonucleotides with 3-bp mismatch (introducing T at positions 3, 4 and 8) as described previously [29].
(TIF)

Figure S3 Temporal changes in Pol II profiles at *edl* and *argos*. Enrichment for Pol II (red; 0–4.7 fold enrichment on a log_2_ scale) across the *edl* and *argos* genes at different time points (min) after Notch activation.
(TIF)

Figure S4 Effect of cycloheximide on Notch response profiles. Graphs show log_2_ fold change in mRNA levels over time (min) for the indicated genes in the presence (black line) or absence (grey line) of cycloheximide (CHX). Error bars indicate standard error of the mean from 3 biological replicates.
(TIF)

Table S1 Summary of differentially expressed genes. Column A, Oligo ID on expression arrays; Column B, FBgn number for each gene; Column C, Gene symbol; Column D,Transcript index; Column E, Transcript CG number; Column F, FBtr number for each transcript; Column G, Transcript symbol; Column H, Chromosome Column I–J, Left and right limits of gene; Column K, Gene orientation: 1 = forward strand, −1 = reverse strand; Column L, Rank by probability of differential expression; Column M, p-value of differential expression; Column N, Q-value of differential expression; Column O, Cluster assignment; Column P, Primary cluster assignment; Column Q, Secondary cluster assignment; Column R, p-value for primary cluster assignment; Column S,p-value for secondary cluster assignment; Column T–Z, PolII class assignment at 0, 10, 20, 30, 40, 60 or 100 min; Column AA–AG,No. of Su(H) binding peaks within 10 kb at 0, 10, 20, 30, 40, 60 or 100 min; Column AH–AK, A values for 4 trials at t = 0 (blue shading); Column AL-BC, Median M values for each transcript at each timepoint (yellow shading).
(XLS)

Table S2 Genes within 10 kb of Su(H) peaks. Tabs indicate Time points. Column headings as follows: GeneFBgn, FlyBase gene identifier; GeneSymbol, Gene symbol; Chromosome, Chromosome; OligoID, Array oligo ID; TransIndex, Transcript number; TransCGNumber, Transcript CG number; TransFBtr, Flybase transcript identifier; TransSymbol, Transcript symbol; TransLeft, Coordinate of left transcript limit; TransRight, Coordinate of right transcript limit; TransStrand, Transcript orientation (1 = 5′ to 3′; −1 = 3′ to 5′); SuHLeft, Coordinate of Su(H) peak left limit; SuHRight, Coordinate of Su(H) peak right limit; MinDist, Minimum distance between transcript and Su(H) peak (0 indicates that the peak overlaps with the transcript).
(XLSX)

Table S3 Su(H) and Hairy binding site analysis. Tabs indicate type of motif analysis. Columns as detailed in each sub-table.
(XLSX)

Table S4 Details of oligonucleotides used for qPCR. Name, gene name and primer orientation; Sequence, Primer sequence.
(XLSX)

Table S5 Time-course expression data. Fold changes in expression for all expressed genes at the indicated time-points (min), results are for individual replicates (rep1-rep4).
(XLSX)

Text S1 Text file with additional details of methods and statistical analysis.
(DOC)
