# Supplementary figures and images for: Transcriptional Dynamics Elicited by a Short Pulse of Notch Activation Involves Feed-Forward Regulation by E(spl)/Hes Genes
Source: PLoS Genet. 2013 Jan 3;9(1):e1003162. doi: 10.1371/journal.pgen.1003162 (PMC3536677; doi:10.1371/journal.pgen.1003162)

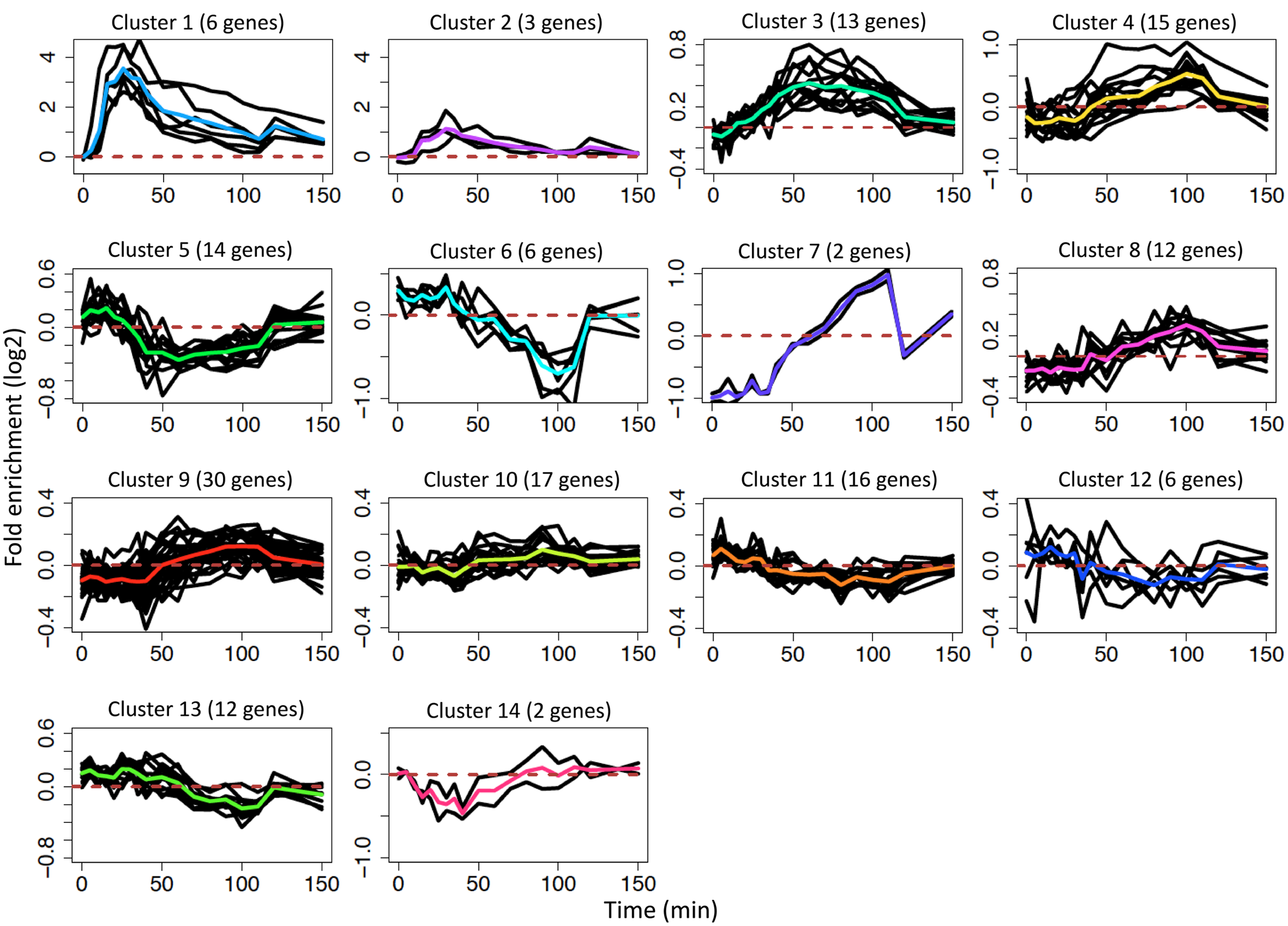

Supplement: Figure S1 — Clustered expression profiles of DE genes. Graphs show log2 fold change in mRNA levels over time (min) for gene clusters. Black lines represent profiles of individual genes and coloured lines show the mean response of the cluster. (TIF) [file pgen.1003162.s001.tif]

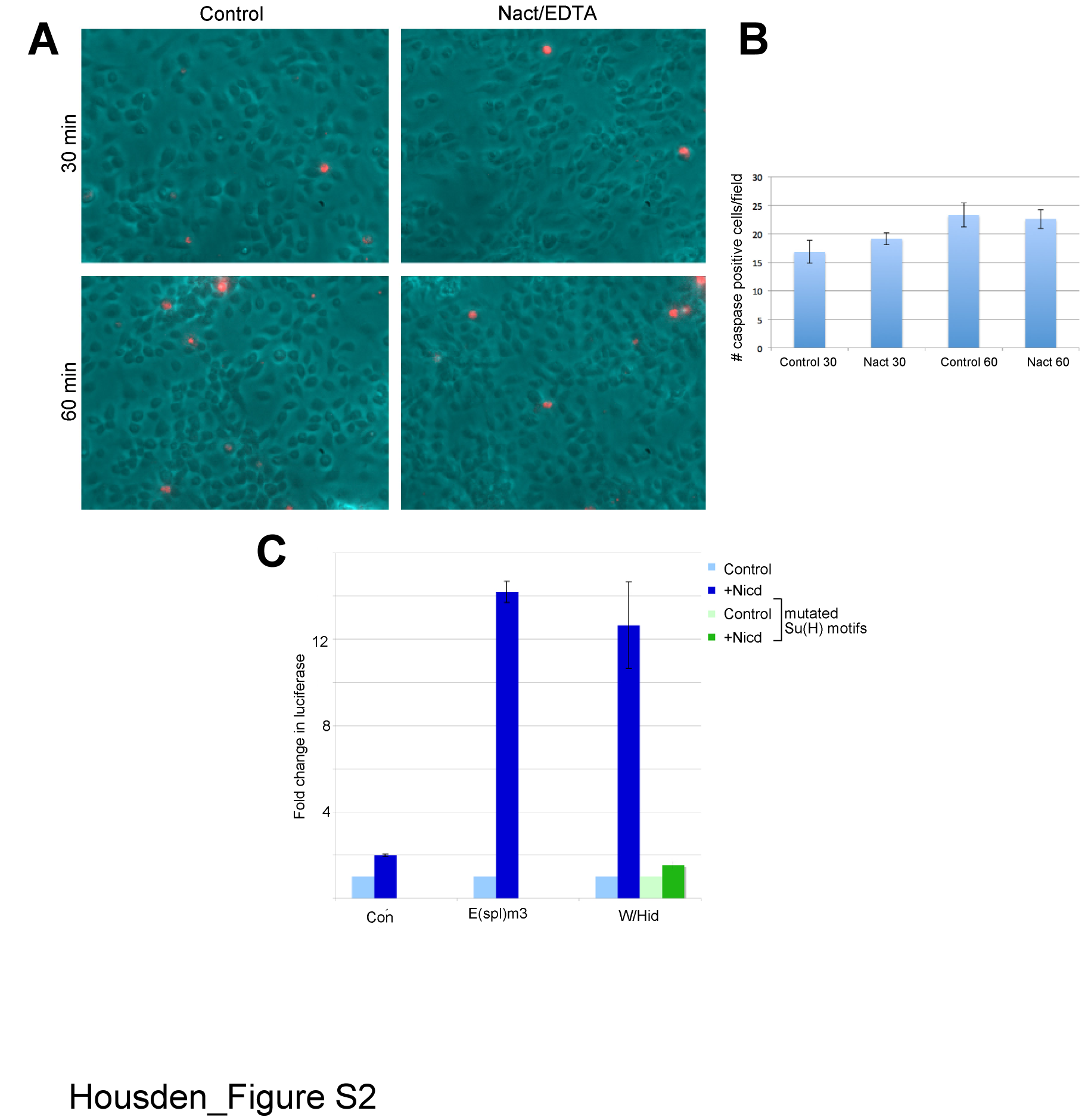

Supplement: Figure S2 — Activated Caspase 3 in treated and untreated cells and role of Su(H) motifs in W/hid enhancer. A. Images show staining for activated Caspase 3 (red) in DmD8 cells at indicated times following a 5 minute pulse of EDTA treatment (Nact/EDTA) or control treatment. The turquoise channel shows a phase contrast image of the field. B. Average number of cells containing activated Caspase 3 per field, quantified from a minimum of 5 fields per condition. Error bars indicate standard error of the mean. No significant differences were found between Notch activated and control conditions (30 min – p = 0.34, 60 min – p = 0.79). C. Response of the indicated enhancers to Nicd in transient transfection assays in Drosophila cells, expressed as fold-change (dark bars) relative to expression levels in the absence of Nicd (pale bars). Mutating Su(H) motifs in the W/hid enhancer (green bars) abolishes responsiveness to Nicd. Error bars indicate standard error of the mean from 3 biological replicates. E(spl)m3, control and un-mutated W/hid luciferase reporters were described previously [29]. Su(H) sites in the W/hid enhancer were mutated using oligonucleotides with 3-bp mismatch (introducing T at positions 3, 4 and 8) as described previously [29]. (TIF) [file pgen.1003162.s002.tif]

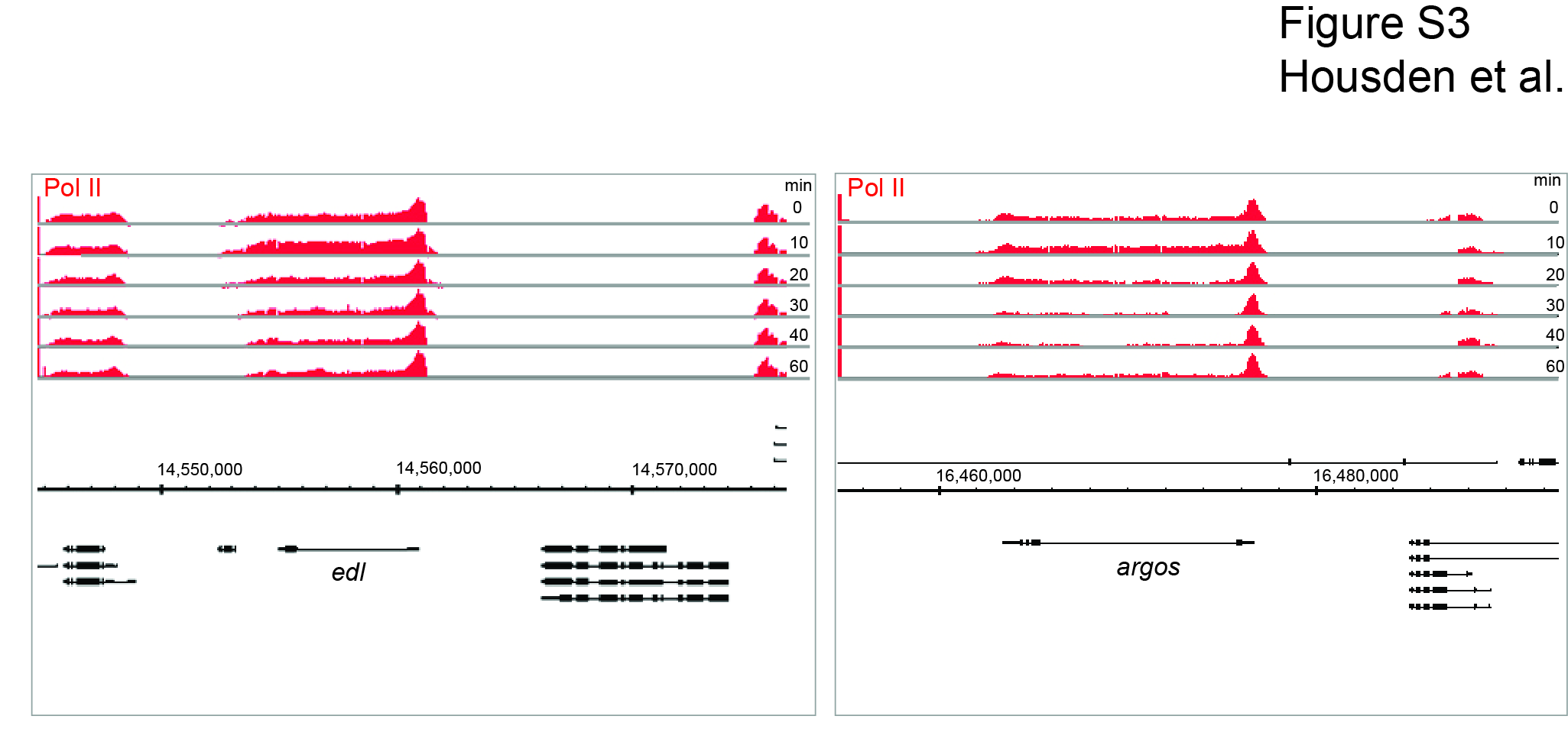

Supplement: Figure S3 — Temporal changes in Pol II profiles at edl and argos. Enrichment for Pol II (red; 0–4.7 fold enrichment on a log2 scale) across the edl and argos genes at different time points (min) after Notch activation. (TIF) [file pgen.1003162.s003.tif]

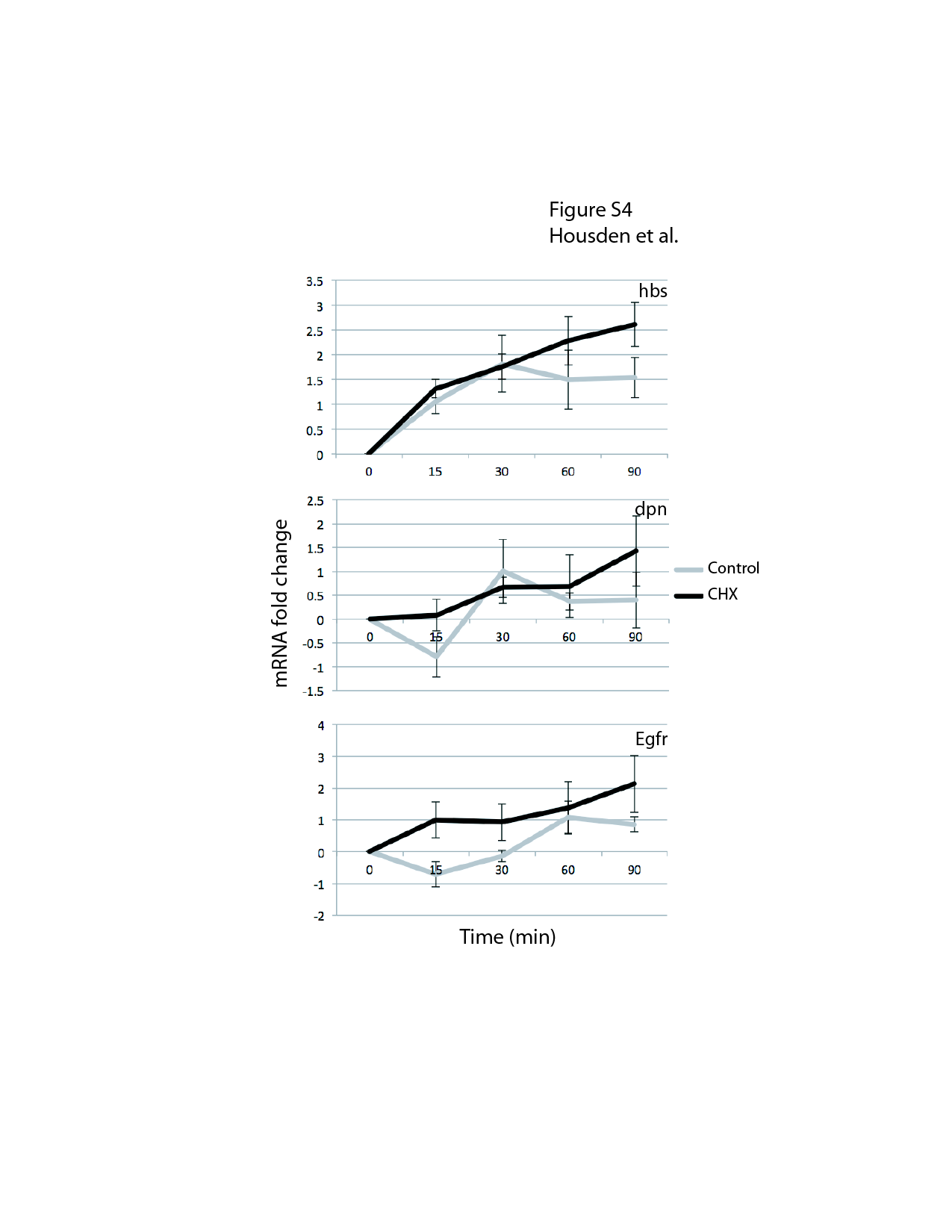

Supplement: Figure S4 — Effect of cycloheximide on Notch response profiles. Graphs show log2 fold change in mRNA levels over time (min) for the indicated genes in the presence (black line) or absence (grey line) of cycloheximide (CHX). Error bars indicate standard error of the mean from 3 biological replicates. (TIF) [file pgen.1003162.s004.tif]
