# Supplementary material for: Transcriptional Dynamics Elicited by a Short Pulse of Notch Activation Involves Feed-Forward Regulation by E(spl)/Hes Genes
Source: PLoS Genet. 2013 Jan 3;9(1):e1003162. doi: 10.1371/journal.pgen.1003162 (PMC3536677; doi:10.1371/journal.pgen.1003162)
Supplement: Text S1 — Text file with additional details of methods and statistical analysis. (DOC) [file pgen.1003162.s010.doc]

**SUPPLEMENTARY MATERIAL**

**LIST OF SUPPLEMENTARY TABLES AND FIGURES**

Table S1: Summary of differentially expressed genes

Table S2: Genes within 10kb of Su(H) peaks

Table S3: Su(H) and Hairy binding site analysis

Table S4: Details of oligonucleotides used for qPCR

Table S5: Timecourse expression data

Figure S1: Clustered expression profiles of DE genes

Figure S2: Activated Caspase 3 in treated and untreated cells and role of Su(H) motifs in *W/hid* enhancer.

Figure S3: Temporal changes in Pol II profiles at *edl* and *argos*

Figure S4: Effect of cycloheximide on Notch response profiles

**SUPPLEMENTARY METHODS**

**RNA profiling using expression microarrays:**

RNA was extracted from cells by removing media and incubating cells in TRI-reagent (Ambion) for 5 minutes. RNA was then purified by extracting with chloroform and precipitating with isopropanol before resuspension in DEPC treated water. DNA was removed by treating with DNase (DNA-free kit - Ambion).

Labelling: for each trial, 50 µg of the untreated RNA sample from each time point was pooled to create a trial specific reference sample. 45 µg of sample or reference pool RNA was mixed with 1.5 µM anchored oligo(dT)23 (Sigma) and incubated at 65°C for 10 minutes. Reverse transcription was performed in the presence of 1nmol fluorescently labelled Cy3- or Cy5-dCTP (GE Healthcare) and 20U RNAsin (Promega) with 400U SuperScript III (Life Technologies) at 46°C for 2 hours. The product was purified using Sephadex G50 columns (GE Healthcare). Samples from trials #1 and #3 were labelled with Cy5 and trials #2 and #4 as dye-swap with Cy3. Co-hybridisation of sample vs reference control to INDAC long oligonucleotide microarrays printed on PowerMatrix slides (Full Moon Bioscience; Flychip FL003; GEO platform accession GPL8244) was performed for 16 hours at 51°C using a GeneTac hybridisation station (Digilab Genomic Solutions Inc). Post-hybridisation washes were performed according to the slide manufacturer’s recommendation. Detailed protocols for array spotting, labelling, hybridisation and washes are available at http://www.flychip.org.uk/protocols/.

Expression array analysis: arrays were scanned using the GenePix 4000B dual laser scanner (Axon Instruments) at 5µm resolution and individually optimised PMT gain settings. Intensity values for each probe were extracted using Dapple . The R package *limma* was then used to normalize the arrays. For each array, the background intensity level of the array image was first adjusted before loess normalization was applied to the MA values of the array. The intensities of the two channels of all 72 arrays (4 replicates at 18 time points) were then quantile normalized, such that the intensities all had the same empirical distribution.

Missing values arise because genes are not expressed or there is poor quality on the array. We systematically removed oligos with missing values at too many time points. First, any oligo having two or more missing values at a given time point was designated “missing” at that time point. Oligos were subsequently removed from further analysis if six or more time points during the first 16 time points (i.e., during the first 110 min) were “missing”. This resulted in 7,467 oligos (from the total of 14,569 oligos printed on the microarray) that had signals indicative of expressed mRNAs. After this quality screen, absent values for the remaining oligos were imputed . Resulting expression values for the corresponding transcripts are summarized in Table S5. Significant differential expression over the time course was then detected using EDGE software ; results are summarized in Table S1.

**Chromatin immunoprecipitation and hybridization to genomic tiling arrays:**

Processing tissue culture cells for ChIP was based on the methods in Birch-Machin et al with modifications as follows : chromatin was cross-linked by fixing cells for 10 minutes with 1% formaldehyde (Sigma) at room temperature before addition of Glycine (Sigma) (0.2M final concentration) to stop the crosslinking reaction. Cells were then detached from plates and washed twice with PBS, keeping samples at 4°C at all times. Chromatin was released from cells by incubation with nuclear lysis buffer (50mM Tris-HCl pH 8.1, 10mM EDTA, 1% SDS, 1X complete protease inhibitor (Roche)) for 10 minutes on ice. Crosslinked chromatin was fragmented by sonication to an average length of approximately 500bp. Chromatin was then pre-cleared by addition of rabbit IgG and protein G agarose beads (Santa Cruz Biotechnology) in IP dilution buffer (IPDB) (20mM Tris-HCl pH 8.1, 150mM NaCl, 2mM EDTA, 1% Triton X-100, 0.01% SDS, 1X complete protease inhibitor (Roche)) and incubation at 4°C for 5 hours with gentle agitation. 2µg Su(H) (Santa Cruz Biotechnology) or Pol II (Abcam) antibody per sample was preincubated with protein G agarose beads in IPDB for 5 hours at 4°C with gentle agitation. Precleared chromatin was enriched by addition of protein G agarose beads with prebound antibody and incubation at 4°C overnight with gentle agitation. Beads with chromatin bound were washed twice with wash buffer 1 (20mM Tris-HCl pH 8.1, 50mM NaCl, 2mM EDTA, 1% Triton X-100, 0.1% SDS), twice with wash buffer 2 (10mM Tris-HCl pH 8.1, 250mM LiCl, 1mM EDTA, 1% NP-40, 1% deoxycholic acid) and once with TE pH 8.0. Chromatin was then eluted from the agarose beads by incubation with elution buffer (100mM NaHCO_3_, 1% SDS) for 10 minutes with vigorous shaking before reversing crosslinks (of precipitated samples and total input) by incubating at 65°C for 5 hours with NaCl (0.27M final concentration). Remaining proteins were removed by incubating with proteinase-K at 55°C overnight. DNA was then purified by phenol/chloroform extraction and ethanol precipitation. For array analysis, DNA fragments and total input samples were amplified by ligation mediated PCR and labelled before hybridisation to tiling arrays.

Labelling: to duplicate reactions the following was added; 1 μg of amplified DNA with 30 µl 2.5X Random Primers Solution (BioPrime DNA labelling kit, Life Technologies), incubated at 100°C for 5 minutes and then cooled on ice. The samples were labelled as technical dye-swap replicates using the BioPrime DNA labelling kit (Life Technologies) in the presence of 4.5nmol fluorescently labelled Cy3- or Cy5-dCTP (GE Healthcare) and 72 U of Klenow at 37°C. After 2 hours, reactions were stopped with 7.5 µl Stop Buffer (BioPrime DNA labelling kit, Life Technologies). Duplicate reactions were pooled into single tubes, precipitated with 165 µl isopropanol plus 0.2M sodium chloride and washed with cold 80% ethanol. The DNA was resuspended in 50 µl water and quantified on a Nanodrop spectrophotometer (Thermo Scientific). 34 µg sample DNA and 34 µg control DNA were combined, dried in a speed vac with medium heat and resuspended in 12.3 µl water. For each hybridisation 29.5µl 2X Hybridization Buffer, 11.8µl Hybridization Component A and 1.2µl Alignment Oligo (NimbleGen Hybridization Kit) were combined and 31.7µl added to each hybridisation sample. Samples were incubated at +95°C for 5 minutes, 42°C for 5 minutes and 41 µl loaded onto the array. The hybridisation was performed to NimbleGen *D. melanogaster* ChIP-chip 2.1M Whole-Genome Tiling Arrays in the NimbleGen hybridisation station at 42°C (mix mode B) for 18 hours. Post-hybridisation washes were performed according to the NimbleGen Wash Buffer Kit instructions.

Tiling array analysis: arrays were scanned using the GenePix 4000B dual laser scanner (Axon Instruments) at 5µm resolution and individually optimised PMT gain settings. Intensity values for each probe were extracted using the NimbleScan software. Results were obtained for 3 replicates at 7 time points (21 arrays). Quantile normalization was applied to the 3 arrays at each of the 7 time points. One replicate at 10 min for Su(H) samples was discarded due to quality issues. Since DNA samples for the first 3 time points and the last 4 time points were collected separately, there was potentially a batch effect on the resulting tiling arrays: this was the case for Su(H) samples, which had generally lower binding signals compared to Pol II samples. We therefore applied batch effect correction to Su(H) tiling arrays.

**Hybrid peak calling method**

The method combined the statistical models in TiMAT (http://bdtnp.lbl.gov/Fly-Net/chipchip.jsp?w=timat) and Tamalpais to assess the uncertainty in probe signals as well as in peak length. The algorithm consisted of two steps:

1. Conversion of the probe signals into binary data. Specifically, we computed the q value and then, using specified FDRs to decide the cutoff for the q value, determined the cutoff for the binary probe signals. The q value calculation requires the p values of individual probes and largely follows the method in TiMAT where the symmetric null p values are computed, assuming that the left half of the empirical distribution of the log_2_ probe signals indicates the noise in the data. The left part of the probe signal distribution is therefore reflected about 0 (or the median, which should be close to 0 after normalization) to obtain a distribution, *F_0_*, symmetric about 0 (or the median), which is assumed to be the null distribution of probe signals.

The observed signal (on the log_2_ scale) of the *i*-th of *S* probes is denoted by *Y_i_.* The empirical null p value of *Y_i_,* denoted by *p*_i_, is then computed with respect to the null distribution *F_0_* as:

*p_i_* = proportion of values comprising *F_0_* that exceed *Y_i_*.

Using Storey’s method , a q value, *q_i_*, was computed for each *p_i_*. The *q_i_* relates to the FDR in the following way:

*q_i_* = Expected number of false positives/ Number of probes with a p value < *p_i_*

The stronger the binding signals compared to the noise, the more probes there will be with small p and q values. As the number of probes with small enough q values is very small relative to the total number of probes on the microarray, a high FDR (40%) can be used at this stage to retain the probes represented as 1 in the binary data.

2. Identification of candidate peaks, defined as runs of ones with a statistically significant length, in the binary data. The approach is based on the extreme value model used in Tamalpais but instead of finding the threshold value for the run length, the p and q values are computed according to the following strategy. Consider *L* probes, each having probability *p* of taking the value 1. Let *R_j_* be the length of the *j*-th run of ones, where *j = 1, …, M*. These *R_j_*s form an empirical distribution of the run lengths, whose right tail corresponds to candidate peaks. An extreme value distribution, which has been used to model the lengths of longest runs of heads in a coin tossing experiment , can be used to capture the right tail of the run length distribution. Specifically, we assume that large standardized *R*s have approximately a Gumbel extreme value distribution with mean 0 and variance 1; that is:

*Z* = (*R* −E(*R*))/SD(*R_j_*) ∼EV(mean=0;Var=1),

where

E(*R*) = log (*L(1-p))/ θ* + γ/*θ* − 0.5,

Var(*R*)= *π^2^* /6θ*^2^* + 1/12,

*θ* = log(1/*p*),

*p* = Pr(a probe is classified as a 1),

in which γ is the Euler-Mascheroni constant (γ = 0.577.... ). An extreme value distribution with mean 0 and variance 1 has the following distribution function:

Pr(*Z* ≤ *z*) = exp { − exp ( −  *(z*−*μ)*/*σ* )}

with location parameter *μ* = −*γ*√ 6/*π* and scale parameter *σ* = √6/*π*. The inverse of the scale parameter, *π*/√ 6, gives the value 1.2825. Using this extreme value distribution, a p value for the length of each run of ones can be calculated and the q values computed using Storey’s q value method .

The above method was used to identify bound regions in both the Su(H) and the Pol II ChIP. For Pol II data, two consecutive rounds of peak calling were performed. First to identify relatively sharp peaks, that corresponded to locations where Pol II was enriched near the transcription start site of a gene (e.g. poised), the criteria used were similar to those for Su(H). Second, to identify the broad regions where Pol II was bound throughout the gene, a more relaxed cut-off was used for the binary probe signals before applying the stringent cutoff in the peak identification.

**Localization of Su(H) binding relative to genes**

Genes neighbouring the Su(H) peak on both strands, irrespective of distance, were identified using a custom-written Perl script to identify the two closest genes on either strand in both directions. These were cross-referenced with DE expressed genes to identify peaks in proximity to DE genes [1]. From this intersection, we determined the distance between the peaks and the nearest DE gene. A 10kb window was sufficient to capture the majority of these associations. Increasing the window beyond 10kb had little impact on the number of Su(H) peaks that could be related to DE genes or on the odds of Su(H) binding being associated with DE, although it remains possible that some very long range associations will have been eliminated (i.e. extending over >2 genes from the peak).

**Defining Pol II binding classes**

The following criteria were used to define the Pol II binding class:

1. UB, if any of the following is true:
   1. no peaks detected 400bp+/-TSS;
   2. a peak overlaps 400bp+/-TSS, but does not cover >60% of 400bp+/-TSS;
   3. a peak overlaps 400bp+/-TSS, but max binding (on log_2_ scale) is <2;
2. P, if all the following are true:
   1. a peak covers at least 60% of 400bp+/-TSS;
   2. max binding (on log_2_ scale) ≥2;
   3. body of transcript (from 400bp downstream of TSS to end of transcript) either has no significant binding or the identified peaks do not cover >60% of body of transcript.
3. AP, if all of the following are true:
   1. peaks identified both near TSS and in body of transcript,
   2. ≥60% coverage of 400bp+/-TSS and body of transcript,
   3. max binding (on log_2_ scale) in 400bp+/-TSS is ≥2,
   4. Stalling Ratio, defined as max binding near TSS / median binding in body of transcript, is ≥2.

Note that both max and median are on log_2_ scale

1. AU, same as AP except that Stalling Ratio < 2

**Regression analysis of relationships between initial Pol II/Su(H) binding and differential expression**

We carried out regression analysis to investigate and quantify the relationships between Pol II and Su(H) binding at 0 min and temporal differential expression of genes. Except where otherwise noted, estimation of the regression models below was performed using *bayesglm* in the R package *arm* .

For the odds of temporal differential expression, we estimated the effects of multiple factors in the following nonlinear regression:

1. Log odds of DE = β_0_ + β_1_Pol + β_2_Su(H) + β_3_Pol×Su(H) + β_4_log(C_0_) + error,

where log is natural log, Pol is a categorical variable with four values (UB, P, AP and AU), Su(H) is binary (binding or no binding; we considered two conditions (i) within 10kb of the transcribed region (ii) within 10kb of transcriptional start site (TSS)) and C_0_ is the mean (across replicates) absolute expression level of a gene/transcript at 0 min. The symbol × indicates statistical interaction between the two factors. Including the interaction term in the regression allowed us to address questions such as “do Su(H) binding and poised Pol II (Pol=P) together increase odds of DE more than Su(H) binding and no Pol II binding (Pol=UB)?”.

By applying model (1) to all expressed genes, we estimated that Su(H) binding and the P state of Pol II have statistically significant, positive effects on the odds of DE: their effects are estimated to be 2.78 and 0.77 respectively. The relatively large effect (2.78) of Su(H) indicates that Su(H) binding is the driving factor of differential expression.

To further investigate the roles of Su(H) and Pol II, we applied the following regression model separately to transcripts either with Su(H) bound within 10kb of the gene or without:

1. Log odds of DE = β_0_ + β_1_Pol + β_2_log(C_0_) + error

Without Su(H) binding at 0 minutes, a paused gene was found to be significantly more likely to differentially express, whereas an AU gene was significantly less likely to differentially express. We estimated that Pol II pausing had a significantly positive effect of 0.78 on average (p value: 2×10^-5^) on the log odds of DE. In other words, Pol II pausing increased the odds of DE by e^0.78^=2.18, compared to the overall average odds of DE. We also estimated that the AU state has a significantly negative effect of -0.47 on average (p value: 0.005) on the log odds of DE, corresponding to a decrease by e^-0.47^ = 0.63 in the odds of DE. In addition, the absolute expression level (on the log scale) also had a significantly negative effect of -0.21 on the log odds of DE.

By applying the same model to genes with Su(H) bound at 0min, we found that the impact of Su(H) binding on the log odds of DE superseded the positive effects of the P class of Pol II binding. In this case, we estimated that the effects of the P class were no longer significant but the AU state remained significant with a negative effect of -1.9. Furthermore, the absolute expression level at 0 min did not appear to significantly impact the odds of DE, when Su(H) was bound near the gene (or TSS).

**SUPPLEMENTARY REFERENCES**

1. Buhler J, Ideker T, Haynor D (2000) Dapple: improved techniques for finding spots on DNA microarrays. UWCSE Tech Report UWTR 2000-08-05, Department of Computer Science and Engineering, University of Washington, Seattle, WA, August.

2. Smyth GK, Speed T (2003) Normalization of cDNA microarray data. Methods 31: 265-273.

3. Fu AQ, Russell S, Bray SJ, Tavaré S Bayesian clustering with the Dirichlet-process prior. Under review.

4. Storey JD, Xiao W, Leek JT, Tompkins RG, Davis RW (2005) Significance analysis of time course microarray experiments. Proceedings of the National Academy of Sciences of the United States of America 102: 12837-12842.

5. Birch-Machin I, Gao S, Huen D, McGirr R, White R, et al. (2005) Genomic analysis of heat-shock factor targets in Drosophila. Genome Biology 6: doi:10.1186/gb-2005-1186-1187-r1163.

6. Johnson WE, Li C, Rabinovic A (2007) Adjusting batch effects in microarray expression data using empirical Bayes methods. Biostatistics 8: 118-127.

7. Bieda M, Xu X, Singer MA, Green R, Farnham PJ (2006) Unbiased location analysis of E2F1-binding sites suggests a widespread role for E2F1 in the human genome. Genome Res 16: 595-605.

8. Storey J (2002) A direct approach to false discovery rates. J R Statist Soc Series B: 479-498.

9. Storey J, Tibshirani R (2003) Statistical significance for genome-wide studies. Proceedings of the National Academy of Sciences: 9440-9445.

10. Shilling M (1990) The longest run of heads. The College Mathematics Journal: 196-207.

11. Gordon L, Shilling M, Waterman M (1986) An Extreme Value Theory for Long Head Runs. Probab Th Rel Fields: 279-287.

12. Gelman A, Jakulin A, Pittau M, Su Y (2009) A Weakly Informative Default Prior Distribution For Logistic And Other Regression Models. The Annals of Applied Statistics 2: 1360-1383.
